# A valid strategy for precise identifications of transcription factor binding sites in combinatorial regulation using bioinformatic and experimental approaches

**DOI:** 10.1186/1746-4811-9-34

**Published:** 2013-08-24

**Authors:** Hailong Wang, Shan Guan, Zhixin Zhu, Yan Wang, Yingqing Lu

**Affiliations:** 1 State Key Laboratory of Systematic and Evolutionary Botany, Institute of Botany, Chinese Academy of Sciences, 20 Nan Xin Cun, Beijing 100093, China; 2 University of Chinese Academy of Sciences, Beijing 100049, China

**Keywords:** *cis* element, MYB, bHLH, EMSA, Dual-luciferase transient expression assay

## Abstract

**Background:**

Transcription factor (TF) binding sites (*cis* element) play a central role in gene regulation, and eukaryotic organisms frequently adapt a combinatorial regulation to render sophisticated local gene expression patterns. Knowing the precise *cis* element on a distal promoter is a prerequisite for studying a typical transcription process; however, identifications of *cis* elements have lagged behind those of their associated *trans* acting TFs due to technical difficulties. Consequently, gene regulations via combinatorial TFs, as widely observed across biological processes, have remained vague in many cases.

**Results:**

We present here a valid strategy for identifying *cis* elements in combinatorial TF regulations. It consists of bioinformatic searches of available databases to generate candidate *cis* elements and tests of the candidates using improved experimental assays. Taking the MYB and the bHLH that collaboratively regulate the anthocyanin pathway genes as examples, we demonstrate how candidate *cis* motifs for the TFs are found on multi-specific promoters of chalcone synthase (CHS) genes, and how to experimentally test the candidate sites by designing DNA fragments hosting the candidate motifs based on a known promoter (*us1* allele of *Ipomoea purpurea CHS-D* in our case) and applying site-mutagenesis at the motifs. It was shown that TF-DNA interactions could be unambiguously analyzed by assays of electrophoretic mobility shift (EMSA) and dual-luciferase transient expressions, and the resulting evidence precisely delineated a *cis* element. The *cis* element for R2R3 MYBs including *Ipomoea* MYB1 and *Magnolia* MYB1, for instance, was found to be ANCNACC, and that for bHLHs (exemplified by *Ipomoea* bHLH2 and petunia AN1) was CACNNG. A re-analysis was conducted on previously reported promoter segments recognized by maize C1 and apple MYB10, which indicated that *cis* elements similar to ANCNACC were indeed present on these segments, and tested positive for their bindings to *Ipomoea* MYB1.

**Conclusion:**

Identification of *cis* elements in combinatorial regulation is now feasible with the strategy outlined. The working pipeline integrates the existing databases with experimental techniques, providing an open framework for precisely identifying *cis* elements. This strategy is widely applicable to various biological systems, and may enhance future analyses on gene regulation.

## Background

The transcription process can be highly dynamic and sophisticated in eukaryotic cells [1], and its initiation typically involves a recognition between a transcription factor (TF) and a *cis* element at the upstream region on a gene. Knowing the precise sequence of a *cis* element for its associated TF is therefore critical for understanding the transcription process of a given gene [2]. The coupling process, however, has not been understood in a balanced way, as *cis* elements have not been clarified for many well characterized *trans* acting TFs.

Both technical and inherent difficulties appear to contribute to the situation. In the previous efforts devoted to *cis* identification [3], a widely taken strategy was segment-dissection. Typically, the 5’ promoter region of a gene was cut by segments and examined how such manipulations affected the expression of the gene, then an inference was made on which segment was likely responsible for the transcription initiation. The protocol can be laborious and frequently result in only rough estimates of *cis* elements in simple cases or inconclusiveness in cases of multiple TFs (such as those in the combinatorial regulation). When the correct segments are identified, their lengths can be inconsistent between reports due to uncertainties about the exact binding sites.

The situation may become inherently more chaotic in some cases. The DNA binding of a TF *per se* may not require a full participation of all nucleotides within the binding region (previously referred to as gapped or degenerate *cis* element [4]) on a promoter, causing more or less varied sequence content and consequently an increased difficulty for identifying the relevant *cis* element. The bordering nucleotide sites around a *cis* element may also influence TF-DNA binding without being part of the TF-DNA complex. More subtly, different species’ versions of the same member of a TF family may recognize somewhat varied *cis* elements due to evolution of the TF-DNA interaction.

It seems lack of precise identifications of *cis* elements cannot be circumvented by the existing approaches. A recent survey on MYB *cis* elements [5] shows that rich experimental data has been collected on MYBs across kingdoms, and many *cis* elements have been reported, but MYB-DNA interactions remain vague. In plants, most MYBs have R2 and R3 domains [6], and the *cis* elements for R2R3 MYBs have been reported in various lengths from 4 (AACA) in the case of rice OSMYB5 by DNase I footprinting analyses [7] to 14 nucleotides (TAT AAC GGT TTT TT) in that of soybean GmMYBs by yeast one hybrid [8]. While these reports do not show coherence in the length of R2R3 binding region, there is no reason to believe that the binding sites for plant R2R3 MYBs should vary greatly in length. In contrast, the R2R3 domains of mouse c-MYB had been shown to bind to AACNG via the nuclear magnetic resonance [9], and the crystallization of protozoan *tv*MYB1-DNA suggested the binding sequence to be a/gACGAT [10]. In other words, the range of R2R3 MYB binding sites has been poorly defined in planta, causing problems for further analysis. Apparently, a more focused identification method is desired for pinpointing the *cis* element for a given TF.

In an analysis of the combinatorial regulation on the flavonoid network, we have developed a strategy that is highly effective in identifications of *cis* elements, particularly for plant systems. The strategy features bioinformatically generated *cis* candidates and their effective validations via experimental means based on some of the existing protocols. The initial step places a bioinformatic analysis in the forefront, which mines candidate *cis* sites species- and locus-wide (assuming a tractable *cis* element). It takes advantage of partially known *cis* information for one of the TFs involved in combinatorial regulation in order to predict the likely *cis* element for another TF in interest. For instance, the binding sites for bHLHs have been known for some members of the TF family. Both mouse c-myc [11] and *Brassica* bHLH [12] recognize CACGTG, which was initially known as the core of the G-box (−TCTTACACGTGGCAYY-) on the promoter of a small subunit of ribulose 1, 5-bisphosphate carboxylase gene [13]. Since the regulation of the anthocyanin pathway requires both MYB and bHLH (there is evidence for the bHLH’s binding to CACGTG [14]), this attribute of bHLH may serve as an anchor for obtaining the *cis* sites for MYB via our bioinformatic approaches.

Regulation by combinatorial TFs features many biological processes. Here we focused on better known MYB, bHLH and WD-repeat protein (WDR) families [15] in plants. These three kinds of TFs may form a complex to influence trichome formation [16] and proanthocyanidin synthesis [17], in additional to anthocyanin synthesis [18]. As part of the flavonoid network, the components of anthocyanin pathway are relatively well-defined, mainly consisting of chalcone synthase (CHS), chalcone isomerase (CHI), flavanone 3- dioxygenase (F3H), flavonoid 3’-monooxygenase (F3’H), dihydroflavonol 4-reductase (DFR), anthocyanidin synthase (ANS), and UDP-glucose flavonoid 3-O-glucosyltransferase (3GT) [19]. These supposed regulons and their TFs have been identified in several species. The MYB-bHLH-WDR complex have been known to include C1- B/R- PAC1 in maize [20,21], AN2 - AN1 - AN11 in petunia [22,23], and MYB1 - bHLH2 - WDR1 in *Ipomoea*[24-26], respectively. Meanwhile, MYB and bHLH (but not WDR) have been known to interact with promoters in order to fulfill their roles in gene regulations. The anthocyanin pathway system is thus an ideal system for examining TF-DNA interactions.

We take *CHS* as an example here, showing that once candidate *cis* motifs are generated from the bioinformatic analysis, their validations may be effectively tested through site-directed mutagenesis and experiments targeting specific TF-DNA interactions. Numerous tests have suggested that electrophoretic mobility shift assays (EMSAs) [27,28] and transient expression assays using living cells [29,30] are highly effective in TF-DNA interaction analysis. For EMSA, we have obtained the best resolution with commercialized fluorescent dyes including SYBR® Green and SYPRO® Ruby (Molecular probes/Life technologies), which may bind to nucleotide and protein, respectively. Sequential applications of these dyes to the same gel and exposures of the gel under different light conditions lead to detections of unambiguous signals of DNA-protein interactions. For dual-luciferase transient expression assays, we have made promoter constructs with desired site-mutations, and engaged particle bombardment and transient gene expressions in living-cells for analyzing candidate motifs. The complete working pipeline is detailed below for precise *cis* identifications on genes under combinatorial regulation.

## Results and discussion

### Bioinformatic searches for candidate *cis* motifs

**Figure 1:**
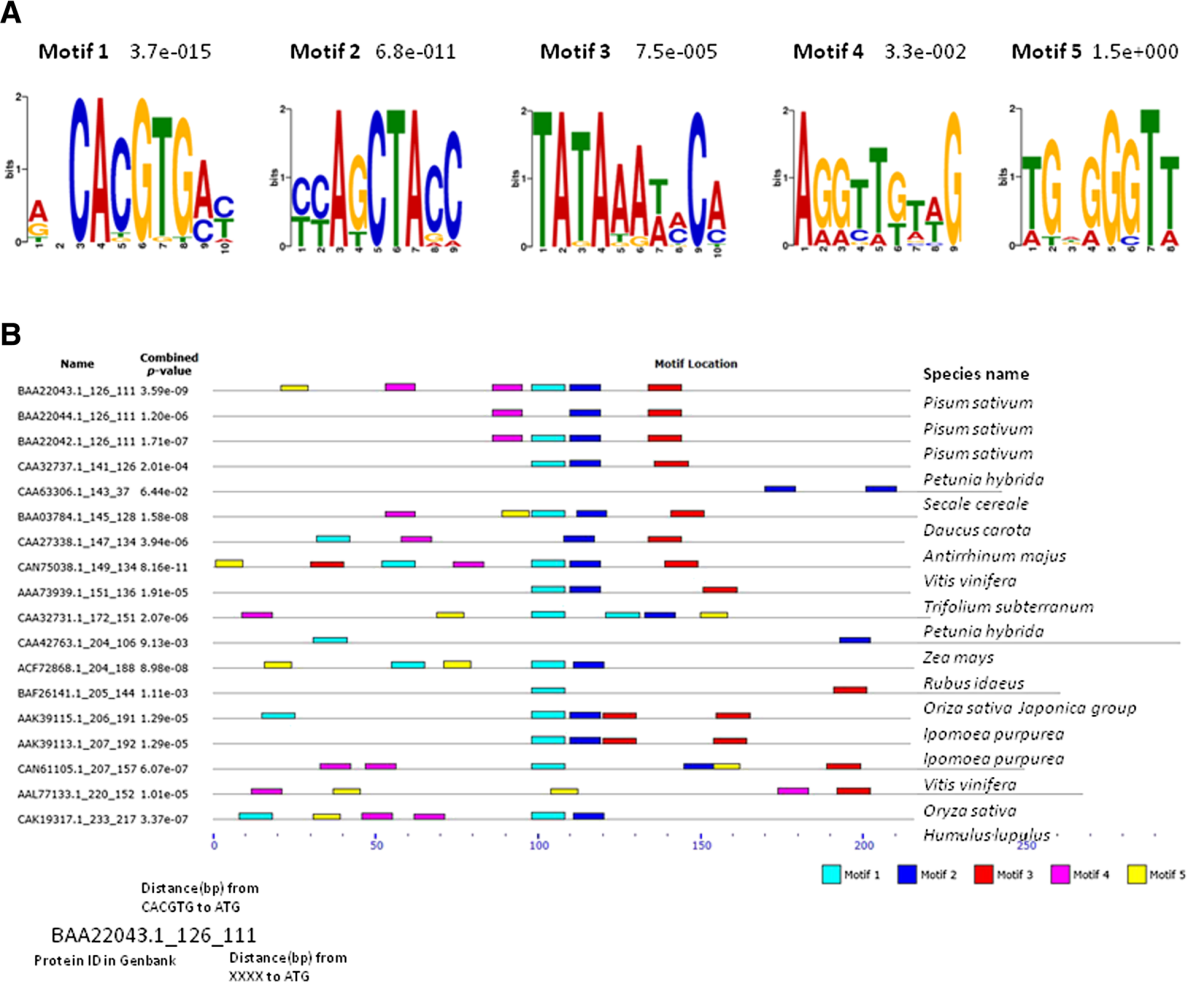
**Bioinformatic searches on promoters of*CHS*. (A)** Sequences and *e*-values of the five motifs detected by MEME (http://www.meme.nbcr.net/meme/) on the 18 *CHS* promoters that show patterns of CACGTG (6–20) CTAC from the bioinformatic searches. The y-axis represents the information content, and the x-axis lists the position of each nucleotide site within the motif. **(B)** Distributions of the five motifs on the *CHS* promoters from 13 species. The sequence label is explained with the sample of BAA22043.1 shown at the left corner, and five motifs are represented by colored rectangles, respectively. The promoter length is scaled from the 5’ end. The combined *p*-value is for the collective presence of all motifs on a sequence.

Our first step was to collect promoter sequences of the regulons for a TF of interest. Since we were interested in MYB recognition sites that governed the combinatorial regulation on the anthocyanin pathway, we carried out bioinformatic searches on promoters of all known anthocyanin pathway genes (Additional file 1). Ten coding sequences of the anthocyanin pathway genes expressed in petals of the common morning glory were taken as templates to perform the searches in the NCBI’s plant databases (gbplnxx.seq.gz). Each search was conducted on one of the templates including *MYB1* (*Ipmyb1*, AB232769), *bHLH2* (*bH2b*, EU032619), *WDR1* (*Ipwd1a*, AB232777), *CHS-D* (*us1,* AF358659), *CHI* (*fl1,* AF028238), *F3H* (*fl1,* U74081), *F3’H* (*purp,* AY333419), *DFR-B* (*fl2,* AF028601), *ANS* (*c,* EU032612), and *3GT* (*b,* EU032615). The ten searches in the NCBI databases led to 571 promoters of anthocyanin pathway genes (Table 1). The dataset was further reduced to contain 271 non-redundant promoters (Additional file 2). We screened these promoters for the presence of CACGTG(…)XXXX via simple Perl scripts (Additional file 1), where X represented any nucleotide and (…) stood for the number of nucleotides separating the two potential motifs (set as 1–50 in our case). Here, CACGTG (allowing one mismatch at each search) was taken as a potential binding motif for the anthocyanin bHLH. Exhaustive searches generated 483 patterns on these promoters. Most of the patterns were not informative, but cases of CACGTG(6–20)CTAC showed promises since these patterns occurred 201 times for 37 promoters of anthocyanin genes and 88 times for 21 *CHS* promoters alone. In particular, 18 *CHS* promoters from 13 species displayed these patterns within approximately 200 bp of the translation starting site. We hence fed the *CHS* promoter sequences to MEME [31] for motif searches. Significant motifs on the promoter region were detected for Motifs 1–3 but not for Motifs 4–5, as the individual *p*-value (≤ 0.05, or 7.5e-005) held only for Motifs 1–3 (Figure 1A). The combined *p*-value was significant for 15 of 18 *CHS* promoters except for one *Rubus* and two *Petunia* sequences (Figure 1B). The same pattern held when four *DFR* promoters from three species were added to the search. In addition to the significant tests, a regular spacing was shown between Motifs 1 and 2 (Figure 1B), and the contents of the Motifs also provided clues. We noticed that Motif 1 contained CACGTG and Motif 3 much resembled the TATA box. Motif 2 was naturally considered the candidate *cis* element for MYB.

**Table 1:** Results of promoter searches using the GenBank data on plants

**Gene_allele**	**Number of promoter sequences**	
	**Before filtering**	**After filtering**
*CHSD_us1*: gi|13774975|gb|AAK39115.1	188	107
*CHI_fl1*: gi|2599056|gb|AAB86474.1	86	13
*F3H_fl1*: gi|1786049|gb|AAB41102.1	24	16
*F3’H_purp*: gi|37694931|gb|AAR00229.1	61	36
*DFRB_fl2*: gi|2599072|gb|AAB84048.1	102	35
*ANS_c*: gi|158515829|gb|ABW69682.1	40	22
*3GT_b*: gi|158714211|gb|ABW79915.1	21	10
*MYB1_a*: gi|97974090|dbj|BAE94388.1	8	4
*bHLH2*_*bh2b*: gi|97974090|dbj|BAE94388.1	6	5
*WDR1_Ipwd1a*: gi|97974146|dbj|BAE94396.1	35	23
Total	571	271

It was no coincidence that the *CHS* promoters in our search presented the Motifs most readily, as available *CHS* promoters greatly outnumbered those of other anthocyanin genes in our dataset (Table 1). Clearly, a large locus-wide promoter sample size may increase the odds of detecting candidates for *cis* elements. The strategy of taking bHLH binding sites as bait is not restricted to the anthocyanin pathway. Many members of the bHLH family are known to interact with other TFs [32], and the bioinformatic search protocol (Additional file 1) is also applicable to cases of TFs with other known *cis* elements. Compared to earlier bioinformatic treatments on *cis* elements enriched via various approaches such as the case regarding *Arabidopsis* anthocyanin regulator PAP1 [33], our analysis is much easier to interpret since a clear biological function is associated with the locus-based searches. More importantly, the candidate *cis* motifs by our protocol do not depend on any prior knowledge of the *cis* to be searched, which distinguishes itself from other bioinformatic methods.

### Validation experiments on the *cis* element for anthocyanin bHLH

**Figure 2:**
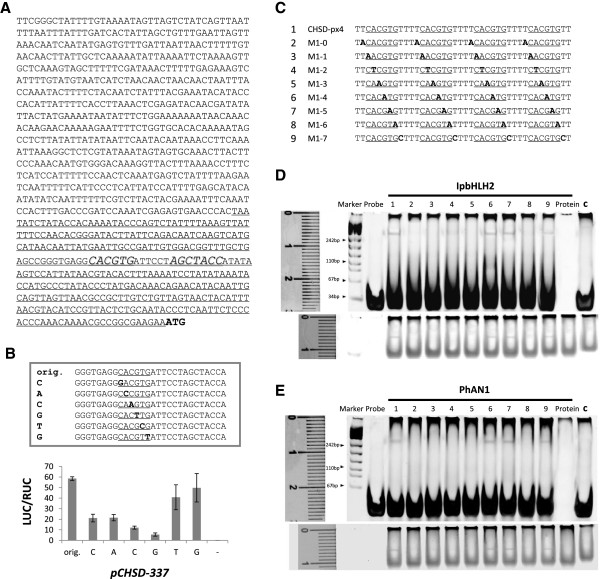
**Experimental tests on Motif 1. (A)** Features of the promoter sequence (1kb) of *CHS-D us1* allele (AF358659) in *Ipomoea purpurea*. The translation starting codon is shown in bold. Motif 1 and Motif 2 are large font and in italic. The underlined is the region built into *pCHS-337*. **(B)** Results of dual-luciferase transient expression activities. The firefly luciferase (LUC) was induced by a 337-nt *CHSD* promoter and effectors expressing the whole coding regions of *MYB, bHLH2,* and *WDR1* of *Ipomoea purpurea,* with renilla luciferase (RUC) as reference. The activity of the native (orig.) promoter (*pCHS-337*) is compared with those of mutated promoters (C, A, C, G, T, G for Motif 1) and a negative control (−, from the tests of the native promoter driven only by the *IpbHLH2* effector). All were measured in the relative fluorescent activities (LUC/RUC), with error bars based on four independent trials. **(C)** Probe sequences (5→3') for the subsequent EMSA binding tests. The probe *CHSD-px4* hosts four dosages of the Motif-1core sequence (underlined) shown on the CHS promoter. Each of the probes (*M1-0→M1-7*) contains a mutation (in bold) at sites of the 5' border, the candidate *cis*, and the 3' border. **(D)** Binding activities of *Ipomoea* bHLH2 (IpbHLH2, EU032618) to the probes above. The upper panel showed the DNA staining of the results, with the loading order followed the probes listed in **(C)**, and the lower panel displayed the protein staining of the same gel. In addition to the DNA marker, the binding result between *CHSD-px4* and IpMYB1 was also loaded in lane c as a control for specificity. The ruler is in unit of cm. **(E)** Binding activities of petunia AN1 (PhAN1, AF260919) to the probes in **(C)** following the same procedures and annotations of **(D)**.

Experimental tests for Motif 1 were conducted on a 1070-bp promoter of the *CHS-D us1* allele (AF358659) that we cloned from *Ipomoea purpurea* leaves (genotype SXHZ1Pa). On this promoter, CACGTG (the core of Motif 1) appears at −205 and AGCTACC in Motif 2 appears −192 from the translation start site (Figure 2A). To focus on these motifs, we constructed a reporter *pCHSD-337* (Additional file 3A) using a 337-nt of the promoter to drive firefly luciferase gene [29]. Meanwhile, we built a reference construct, placing renilla luciferase (RUC) gene under a 35S promoter (Additional file 3A). From the same vector, we made three effector constructs (Additional file 3B) that relied on the 35S promoter to express each of the coding regions of MYB1 (AB232769), bHLH2 (EU032618), and WDR1 (AB232777). A *baichou* strain of *Ipomoea nil* from traditional Chinese herbs was found to be a *WDR1* mutant (KF384189) in our laboratory. The strain shows little expressions of the combinatorial TFs, providing a suitable system for analyzing the TFs’ expressions. In a typical experiment, we simultaneously introduced five kinds of vectors via particle bombardment into the strain’s young leaves. We recorded two fluorescent levels of the reporter proteins, and took their emission ratio (LUC/RUC) on the same cells as a measure of the promoter activity. Combining this experimental setup with site-directed mutagenesis, we detected and compared the contributions of individual nucleotides of CACGTG to the promoter activity. The results suggest that except the last nucleotide, all mutations led to reduced activities of the promoter, with the first 4 sites having the strongest effects under the design of the site mutations (Figure 2B) and the fifth site having the weakest effect. Little background interference was found using the reporter systems.

Since Motif 1 appeared on target for the MYB1-bHLH2-WDR1 combinatorial regulation in living cells, we further examined Motif 1 in EMSAs to make sure that it was bHLH2 that specifically bound to the *cis* sites. To this end, we designed series of probes from the *CHS-D us1* promoter region hosting Motif 1. Besides the local sequence, probes also included mutated sequences at Motif 1 and those at the 5’ and 3’ bordering sites (Figure 2C). We prepared the bHLH2 protein with the pMAL system (NewEngland BioLabs), which used maltose-binding protein (MBP) to tag a protein (Additional file 4). The combined protein was then separated through an amylose resin-based column. Since a whole bHLH2 could not be properly expressed and folded in the *E*. *coli* system, its partial expression was adopted to include the intact bHLH binding domain and the immediately adjacent 3’ conserved region. Including the 3’ conserved region has been shown effective previously [14] and in our tests. To increase the binding signals, target mutations were duplicated twice on the probes. The duplications had been examined in our prior tests to make sure it enhanced signal intensities only without compromising the binding patterns (data not shown). After each probe was exposed to the bHLH2, we examined the TF-DNA interaction on a nondenaturing polyacrylamide gel. If the probe binds to the protein, the TF-DNA complex will move slower than the free probe and protein inside the gel, causing a slower band visible under light for viewing a DNA or protein staining. Since a positive binding can be detected by independent fluorescent signals on the same gel, the evidence for a DNA-protein interaction is more convincing than one of the competition design in previous EMSAs. Here, we observed a positive binding of the MBP-bHLH2 to a probe containing quadruple CACGTG (Figure 2D). Mutations at the 5’ bordering site and the first three and last nucleotides of CACGTG significantly reduced the binding activity while mutations at the fourth and fifth nucleotides of CACGTG and 3’ bordering site did not significantly affect the binding pattern. To make sure of the results, we also tested petunia AN1 (AF260919) and witnessed the same pattern (Figure 2E). The G-box sequence (CACGTG) was hence considered the bHLH recognition element (BRE) for the tested TFs.

In summary, both EMSA and transient expression experiments agree that BREs for the anthocyanin pathway bHLH take the form of CACNNG(T). For the *cis* structure, the fourth (allowing G → A but not G → C) and fifth positions may tolerate certain changes, and the last site prefers G or T. Here, we did not examine all likely substitutions at the 5’ adjacent site to CACGTG since it may be specific to an interacting TF, as we will see later with the case of MYB-DNA interactions.

### Verification and delineation of *cis* motif for anthocyanin MYB

**Figure 3:**
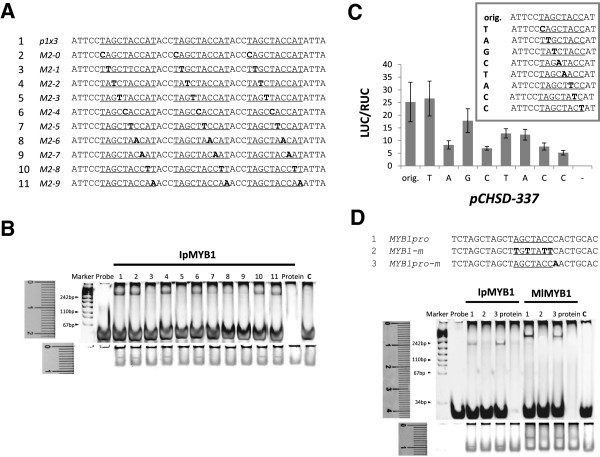
**Experimental tests on Motif 2. (A)** Probe sequences (5 → 3’) for the EMSA tests below. The *cis* candidate Motif-2 (underlined) on the *CHS-D us1* promoter was arranged in triplets (*p1x3*), and mutated sequentially in each of the probes (*M2-0* to *M2-9*) at the sites in bold. **(B)** EMSA results on the binding activities to the probes of (A). The reactions were separated on a 12% SDS-PAGE gel, and shown by the DNA staining (the upper panel) and the protein staining (the lower panel) sequentially. **(C)** Results of dual-luciferase transient expression assays for Motif 2. The mutated promoters were labeled as T, A, G, C, T, A, C, C, whose activities were expressed as LUC/RUC. The original promoter (orig.) was included as a positive control with all three effectors presented, and the case of the promoter plus *IpWDR1* and *IpbHLH2* effectors (−) was shown as a control for the effect of IpMYB1. The error bars were based on four independent trials. **(D)** Binding tests of two MYB1 proteins on an *IpMYB1* promoter (KC794943). The *cis* candidate (in bold) and its two mutated versions were designed on three probes (1–3), respectively. Their individual reactions to IpMYB1 and MlMYB1 were shown on the gel, with the **c** lane including the result of a binding test between IpbHLH2 and *MYB1pro* as a control for specificity. The gel setting followed those of **(B)**.

The *in vitro* confirmation of CACGTG is encouraging, as it concords with our bioinformatic analysis. We can now focus on the binding sites for the anthocyanin pathway MYBs. As mentioned above, reports on *cis* elements for the R2R3 MYBs have been incoherent so far. Maize C1, for instance, was shown to bind to TAACTG of *Bz* (*3GT*) gene promoter by transient expression [34], but to ACCTACCAACC of *A1 *(which encodes DFR) promoter by gel retardation and DNA footprinting [35]. Lesnick and Chandler [36] later proposed a consensus sequence TTGACTGGnGGnTGCG as the binding site for C1. Recently, MYB10 in apple was shown to recognize ACTGGTAGCTATT [37]. These MYBs have been positively characterized as the anthocyanin regulator in the respective species, but their associated *cis* elements reported so far do not agree well with one another. Here, as Motif 2 has been shown to be the likely binding sites for anthocyanin MYBs in our analysis, it was tested on the probe series that arranged Motif 2 in triplets (Figure 3A). MYB1 from *Ipomoea purpurea* (IpMYB1, AB232769), known to regulate the anthocyanin pathway [24], was fully expressed for the EMSA assays (Additional file 4). The binding tests clearly indicated that IpMYB1 bound to the motif by the range of AGCTACC, as mutations outside the region did not alter the binding capacity significantly (Figure 3B). AGCTACC was subsequently considered the putative MYB1 recognition element (MRE). To make sure that the MRE and the BRE were on targets, we mutated both CACGTG and AGCTACC on the promoter *CHSD-337*, and observed little promoter activity (0.68%, se 0.10%) accordingly in dual-luciferase expression assays.

Transient expression experiments were also examined for their effectiveness in delineating and verifying *cis* elements. We constructed reporters with the promoters mutated around the MRE and performed the living cell tests using *pCHSD-337* (Figure 3C). The results confirmed that mutation at the 5’ bordering site did not affect the promoter activity, agreeing with the previous EMSAs on the 5’ border of the *cis* element. Our delineated *cis* element (AGCTACC) resembles ACCTACCAACC in maize [35] at the 5’ side, and agrees with the middle portion of ACTGGTAGCTATT in apple [37] in the reverse complement (GGTAGCT). These previously inconsistent results now converge with our MRE due to the precise identifications presented here.

The IpMYB1 based analysis was examined again in a different species to know whether or not IpMYB1 homologues can recognize the *cis* element identified in *Ipomoea*. We cloned *MlMYB1* (KC794950) from Mulan magnolia (*Magnolia liliflora*), whose amino acid sequence is 49% identical to that of IpMYB1. When the two MYBs were independently incubated with the same probes based on the promoter of *IpMYB1* (KC794943), we found essentially the same binding pattern with only varied binding intensities (Figure 3D). MlMYB1prefered the first probe more than the third one, though the two probes differed only at the nucleotide adjacent to the MRE, while IpMYB1 seemed to favor the third probe. The results suggest that the bordering site of a *cis* element may cause different DNA-binding affinities between TFs.

### MYB-DNA interactions on previous anthocyanin promoters

**Figure 4:**
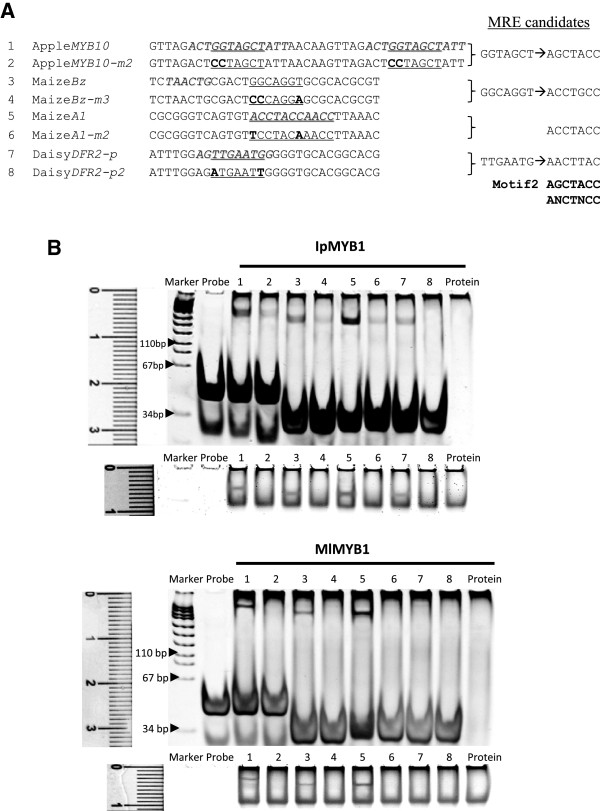
**EMSA results on previous MYB-binding fragments. (A)** Sequences of the probes (5’ → 3’) designed from previous reports. Each pair consisted of one probe based on a reported promoter segment and the other mutated at sites in bold. All predicted *cis* elements from this study were underlined, in comparison to the previously identified *cis* region (shaded and in italic). The first probe hosted duplicated *cis* fragments previously for apple MYB10. **(B)** EMSA results between the probes of (A) and two expressed MYB1 proteins. The binding patterns of IpMYB1 and MlMYB1 to these probes were shown on two gels (all 12% SDS-PAGE). The DNA marker showed the DNA staining (the upper panel) and the ruler (cm) on the same gel measured the protein staining (the lower panel).

Since several different promoter fragments of anthocyanin genes have been previously reported to be the binding regions for anthocyanin MYBs, we extended our DNA-TF tests with IpMYB1 and MlMYB1 to these cases. The purpose was to see whether or not these MYBs could recognize the DNA fragments and better define the length of the associated *cis* elements. Three sets of promoters were examined, including those of Apple *MYB10*[37], maize *A1*and *Bz*[34,35], and African daisy *DFR2*[38]. Though little sequence consensus was visible among the reported binding regions, we found presences of the predicted *cis* elements (underlined) on all of the promoter fragments (Figure 4A). Our tests suggested that IpMYB1 could bind to the DNA fragments at the predicted *cis* sites, as mutations at these sites severely reduced the binding activities. Similarly, MlMYB1 could interact with the same set of probes, showing one exception on the *Gerbera DFR2* promoter (Figure 4B). Once we clearly defined the *cis* element candidates on these previously considered binding regions, we observed a consistency between these candidates and the MRE on the *Ipomoea CHS* promoter (Figure 4A). For the *Gerbera DFR2* promoter, the *cis* element was predicted here to be TTGAATG (AACTTAC), which differed from ANCTNCC noticeably at the sixth site. While IpMYB1 could bind to it at a reduced intensity, MlMYB1 failed to recognize the probe. The recognition capacity appears to vary between TFs for the same *cis* element.

By taking the combinatorial regulation on the anthocyanin pathway as an example, we have demonstrated the validity of the whole strategy for precisely identifying *cis* elements on a relevant promoter. It is evidently not species- or tissues- specific. Promoters of anthocyanin genes (*CHS*, *MYB1*, *DFR*, *3GT*) across species (*I. purpurea*, *Zea mays*, *Malus domestica*, *Gerbera hybrida*) have been successfully scrutinized for their *cis* elements. TFs across species (*I. purpurea*, *M. liliflora, P. hybrida*) have been tested for their binding capabilities. Among the previous tools such as crystallization, DNA footprinting, yeast one-hybrid, EMSA, and transient expressions in *cis* research, we found the combination of site-directed mutagenesis with enhanced EMSA protocol to be the most cost-effective and time-efficient in defining a *cis* element. As the evidence gathered from the EMSA was for physical binding outside the cell, we devised the second system with mutagenesis/particle bombardment/transient expression to collect relevant evidence inside living cells. Obviously, further species-specific *in vivo* approaches including transgenic techniques can be integrated to strengthen the *cis* analysis. The strategy outlined here is versatile enough to accommodate additional techniques of the future.

## Conclusions

With ever increasing genomic data and promoter sequences, bioinformatic searches will offer an indispensable summary of the available data and prove effective in establishing candidate motifs for tractable *cis* elements, about which no prior information is required. The hypothetical motifs can be experimentally tested and delineated with properly designed site-directed mutagenesis and *in vitro* and *in vivo* approaches. This logic is broadly applicable to biological systems. The application will hopefully facilitate both gene functional analysis and gene regulation at large.

## Methods

### Bioinformatic analysis

We performed a blastp search on the Genbank database (http://www.ftp.ncbi.nih.gov/genbank/, up to 10 April 2009) to assemble protein homologs for each of the ten anthocyanin gene templates, using the criteria of sequence identity > 40% and length coverage > 80%. The searches led to a collection of the DNA sequences (n = 571) that were at least 200 bp from the ATG start codon at the 5’ region. We then applied clustalw (http://www.ebi.ac.uk/Tools/msa/clustalo/) to the sequences and only picked the longer sequence for each of the clusters that scored higher than 80% in identity. This process resulted in a non-redundant sample (n = 271) for the subsequent data analysis. Assuming that the binding site of bHLH2 took the form of CACGTG and allowed one mutation at any of the sites on the collected promoters, we searched on both directions for motifs of 4-nucleotides (XXXX) that satisfied CACGTG(…)XXXX under the conditions that (1) the occurrence frequency of each combination > 4 and (2) the distance between CACGTG and XXXX ≤ 50 bp. Since a given combination of CACGTG and XXXX may have slightly varied separating distances, we classified them using the nearest neighbour method [39] to reduce scattering of a particular combination. For each of the classified combinations, we dissected out 100 bp promoter sequences flanking the sites (Additional file 1), and prepared the dataset in fasta format for MEME (http://www.meme.nbcr.net/meme/, version 4.3.0). The parameters for MEME were set as: occurrences of a single motif among the sequences: any number; minimum width of each motif: 6 bp; maximum width of each motif: 10 bp; Maximum number of motifs to find: 5; Search: given strand only. Results for each combination of CACGTG(…)XXXX or XXXX(…)CACGTG were visually checked for likely patterns of motifs, which led to patterns of CACGTG(6–20)CTAC found on multiple promoters.

### Plant materials

The common morning glory (*Ipomoea purpurea*) seeds were planted in the fields of the Botanical Garden of the Institute of Botany, Chinese Academy of Sciences, in Beijing, to provide petals and young leaves for TF and promoter amplifications and cloning. Mulan magnolia (*Magnolia liliflora*) inside the botanical garden provided floral tissue for MYB cloning. Petals of petunia R27 (kind gift from Dr. Quattrocchio) in a growth chamber were used for AN1 cloning. Plants from the ivory seeds of Japanese morning glory (*Ipomoea nil,* strain *baichou*) purchased locally provided leaf materials for the transient expression assays. For consistency, the assays were conducted on only the second young leaf (about 3 cm in diameter) of each plant stem in a growth chamber.

### Promoter sequencing and cloning

The promoter of *CHS-D* (AF358654-9) was obtained from leaf genomic DNA via standard PCRs. The promoter of *MYB1* was isolated via thermal asymmetric interlaced - PCR [40] by specific and random primers. Three specific primers were used for obtaining the *IpMYB1*promoter, including ltMyb + 131 (gctctaaagggaactaggtgccat), ltMyb + 56 (acgatggactccagtccgaccaagcaccctttctcact), and ltMyb + 30 (cggagaccaccttgcagaagaattaacc).

### Dual-luciferase assays

The coding sequences of *MYB1*, *bHLH2*, *WDR1*, firefly luciferase gene and renilla luciferase gene were integrated into pJIT163 at appropriate restriction sites (Additional file 3). Plant transformations by particle bombardment (BioRad PDS-1000/He equipment) were performed on young leaves of the *WDR1* mutant with the abaxial side facing up on the solidified water medium (0.7% agar). Effector and reporter plasmid DNAs each in 1 mg were mixed with 0.2 mg of reference plasmid pJIT163-35S: RUC. Microparticles (1.0 μm, Bio-Rad) were prepared at the concentration of 50 mg/ml, and mixed with prepared plasmids at 2.0 μl microparticles per 1.0 μg plasmid DNA. The particle-DNA complex was then mixed with 2.5 M CaCl_2_ and 0.1 M spermidine, and centrifuged to remove the supernatant. After washing in 70% and 100% ethanol successively, the particle-DNA complex was re-suspended in appropriate amount of 100% ethanol (10 μl for one shot). The leaves were subjected to bombardment at 1100 psi and 26 mm Hg vacuum, and kept in dark at 24 C for about 24 hrs. The luciferase activities were detected by a Glomax 20/20 luminometer following the manufactory’s instruction on Dual-Luciferase® Reporter (DLR™) Assay System (Promega).

### Protein expression and purification

Constructs were built from pMAL-c2G (Additional file 4), as instructed for the pMAL™ protein fusion and purification system (New England Biolabs). The host strain *E*. *coli* BL21 (DE3) was transformed by the target vector and grown at 37 C in LB solution with ampicillin (50 μg/ml) and chloramphenicol (50 μg/ml), and added with IPTG to 0.5 mM when the solution reached 0.5 at OD600, then cultured under 25 C for six hours before protein extraction. One liter of the cultured cell solution was cleaned twice with the loading buffer then ultrasonicated at 400 W in pulses of 5 second for 5 min in ice and centrifuged at 4 C at 12000 rpm for 15 min to collect the supernatant. Affinity-based purification of the proteins was performed on an amylose resin column following the standard protocol, and the separated samples were examined with 12% SDS-PAGE electrophoresis to pick ones with the desired protein expression. Qualified samples were quantified via Bradford method using standard bovine gamma globulin following the manufactory’s instructions on Bio-Rad protein assays.

### Electrophoretic mobility shift assays (EMSA)

Two complementary oligonucleotides synthesized by Sangon (Sangon Biotech) were annealed at 95°C for 10 min in 0.2 × SSC buffer (3 mM sodium citrate, 30 mM NaCl, pH 7.0) then slowly cooled in three hrs to room temperature to make a probe. Each probe was quantified with known quantity of λDNA (Life Technologies) using Picogreen in the quantification module of Rotor Gene 3000 (Corbett Research).

A 10 μl binding reaction mixture, containing 2 μl 5× binding buffer [25% glycerol, 40 mM MgCl2, 5 mM EDTA, 5 mM DTT, 250 mM NaCl, and 50 mM Tris–HCl (pH 7.5)], 20 pmol probe, and 2 μg MBP- target proteins, was incubated at 25 C for 10 min. Samples loaded to a 12% (wt/vol) nondenaturing polyacrylamide gel were subject to electrophoresis in 0.25X TBE buffer at 130 V for 1 h. The gel was covered in 1XSYBR Green EMSA stain (Life Technologies) for 20 min at room temperature in dark and washed twice with dd H_2_O, then visualized for the presence of DNA using a G-Box ChemiXL Fluorescent Imager (Syngene) under 300 nm excitation and 500 - 600 nm emission. The gel was further treated in SYPRO Ruby EMSA stain (Life Technologies) and destained in 10% methanol and 7% acetic acid overnight. Before acquiring its image, the gel was washed twice, then detected for the presence of protein under 300 nm excitation and 550 to 640 nm (EtBr/UV Mid pass filters) emission settings.

## Competing interests

The authors declare that they have no competing interests.

## Authors’ contributions

HW expressed the proteins and collected the EMSA data, SG conducted the bioinformatic searches and wrote the Perl scripts, ZZ collected promoter sequences and performed the dual-luciferase assays, YW participated in an early phase of the study, and YL designed the study and wrote the manuscript. All authors read and approved the final manuscript.

## Supplementary Material

Additional file 1 Bioinformatic searches (instructions with scripts). Click here for file

Additional file 2: Table S1 A list of 271 promoters used in the bioinformatic analysis. Click here for file

Additional file 3: Figure S1 Vector constructions in transient expression assays. **(A)** Construction of the reference and reporter vectors. The fluorescent protein-coding regions are in green. The tested promoter is shown in blue arrow. **(B)** Construction of the effector vectors. The TFs’ coding frames are shown in blank arrows. Click here for file

Additional file 4: Figure S2 Constructs and protein expressions for the EMSA experiments. Figure S2. Constructs and protein expressions via pMAL-c2G system for the EMSA experiments. **(A)** IpbHLH2 (EU032618). **(B)** PhAN1 (AF260919). **(C)** IpMYB1 (AB232769). **(D)** MlMYB1 (KC794950). **(E)** Expressed portions of the bHLHs in amino acid sequences. An EMSA test utilized the target protein expressed along with the maltose –binding protein (MBP). Click here for file
