# Supplementary material for: A valid strategy for precise identifications of transcription factor binding sites in combinatorial regulation using bioinformatic and experimental approaches
Source: Plant Methods. 2013 Aug 24;9:34. doi: 10.1186/1746-4811-9-34 (PMC3847620; doi:10.1186/1746-4811-9-34)
Supplement: Additional file 1 — Bioinformatic searches (instructions with scripts). [file 1746-4811-9-34-S1.doc]

Additional file 1

**Bioinformatic searches**

**I. Collection of promoter sequences of regulons (taking the anthocyanin pathway genes as examples)**

We harvested promoter sequences from Genbank-based databases in the following steps:

1. Download data files of Genbank to a local computer and choose protein sequences for CDs.

**usage: ParseGBgz4Prdct.pl GenbankFileList Output**

GenbankFileList is the file name list of all gzip files downloaded from Genbank; Output saves protein sequences of CDs in fasta format.

1. Query the 10 protein sequences which are translated from the 10 genes of the anthocyanin pathway (see table 1 for details) against the protein sequences collected from step 1 with blastp [1].

**usage:Blastp.pl Query Database Output**

Query saves the 10 protein sequences translated from the anthocyanin pathway in fasta formt; Database is the output of ParseGBgz4Prdct.pl. Except the evalue as <1e-4, the other parameters for blastp are set by default.

1. From the blastp output, proteins which are potentially homologous to the 10 genes of the anthocyanin pathway are obtained under the conditions: Identity percentage≥40%, and the total length of alignment is ≥ 80% length of both query and subject sequences.

**usage:ParseBlastp4Hmlg.pl Blastp.Output Query.Fasta Subject.Fasta Threshold Output** (identity≥40)

Blastp.Output is the output of Blastp.pl; Query.Fasta and Subject.Fasta correspond respectively to the query and subject file of step 2; Threshold formulates the shortest requirement for sequence alignment. The ratios of total alignment length to length of query and subject sequences should be greater than Threshold. In the actual data processing, we set Threshold=0.8; Output collects ids of proteins that are potentially homologues to query sequence.

1. Based on protein ids obtained in step 3, Genbank data files are processed again to choose target nucleotide sequences.

**usage:ParseGBgz4Prmtr.pl HomologFile gbpep.fasta Least Last**

HomologFile is the output of step 3. gbpep.fasta is the output of step 1; in addition to protein sequences, it also includes information about which protein stored in which Genbank file; Least and Last specify respectively the shortest and longest length of promoter sequence (In our data processing: Least=200, Last=2000). This script outputs 10 files that correspond respectively to the 10 genes of the anthocyanin pathway. In each file, promoter sequences of genes that are potentially homologues to a particular gene of anthocyanin pathway are saved in fasta format.

1. For each promoter sequence file obtained in step 4, multiple sequence alignment is performed by clustalw [2].

**usage: clustalw -INFILE=X.prmtr > X.clu**

**usage: CluScoreD.pl X.clu ScoreD**


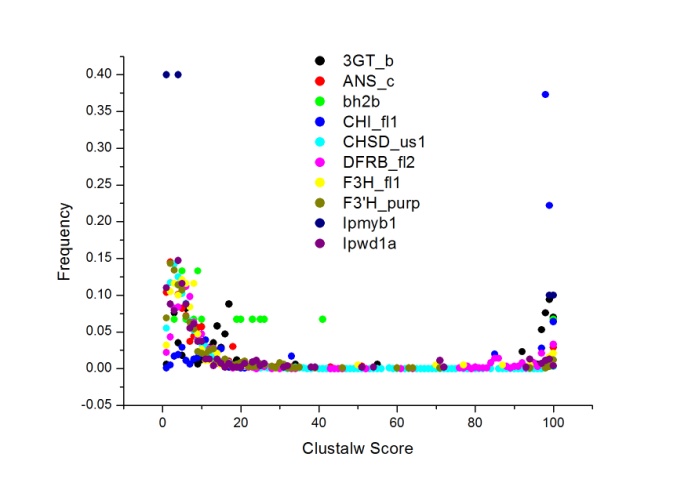
The 10 files obtained in step 4 are imported to clustalw one by one, and 10 outputs of clustalw are saved separately. For each output of multiple sequence alignment, we summarize the pairwise sequence similarity score, and save the distribution in ScoreD.

The figure shows that the majority of promoter sequences fall into two groups: one has similarity scores from 80 to 100; the other has similarity scores ranging from 0 to 40.

1. Based on the similarity score distribution, promoter sequences in one species with high similarity score are regarded as redundant and should be trimmed. In the example given, For each pair of promoter sequences, whose similarity score >=80 and belongs to same species, only the longer one is remained; and this process is continued until the similarity score between any pair of promoter sequences in same species is less than 80.

**Usage：CluMerge.pl Clustalw.output Threshold Remained**

Clustalw.output is output of step 5; Threshold provides the cutoff similarity score to two promoter sequences; Remained saves promoter sequences without redundancy.

**II. Prediction of DNA binding sites (taking bHLH2 (*bh2b*) and MYB1 (*Ipmyb1*) as examples)**

1. The protein sequence of *bh2b* may be queried against transcription factor database of Jaspar[3] with blastp to gather information on likely DNA binding sites of bh2b, which is based on the PWMs of transcription factors that are similar to bh2b. The blastp result shows that bh2b is similar with transcription factors CBF1, MAX, Mycn, USF1, Ahr, ARNT, Myc and RTG3, whose consensus of DNA binding sites recorded in their PWMs is “cacgtg”. Therefore, we initially assign “cacgtg” as the DNA binding sites of bh2b.

The same search is performed on *Ipmyb1*, but no clear consensus can be draw from Jaspar database. The following search is based on the assumption that the binding sites of two collaborative TFs are not far apart (e.g., < 50 bp).

1. Count appearance number of pattern “CACGTG(…)xxxx” and “xxxx (…)CACGTG” in each structure gene’s promoter sequences (multiple times appeared in one sequence is regarded as one appearance). “CACGTG” represents oligos which have at most one nucleotide mismatch with “cacgtg” (all possible oligos are 4X6); “xxxx” represents oligos composed by 4 nucleotide (all possible oligos are 4^4^); “(…)” represents distance between “CACGTG” and “xxxx” that varies from 1 to 50. Patterns whose appearance number is greater than certain times (4 in our actual data processing) are clustered by the nearest neighbour method according to distance variation (in the actual data processing, if distance variation of CACGTG(m)xxxx and “CACGTG(n)xxxx” is less than 10, they are merged together and represented by CACGTG((m+n)/2)xxxx).

**usage:NearestCluster PrmtrFasta Word1 Word1TH Word2 Word2TH ClusterTH AtLeastPrmtrN PatternD**

PrmtrFasta saves promoter sequences in fasta format; Word1 and Word2 represent respectively the 1st and 2nd oligo of a pattern; Word1TH and Word2TH are the lowest scores of pattern match for Word1 and Word2, respectively. One nucleotide match adds 1 to score; ClusterTH is the biggest distance variation for the nearest neighbour clustering; AtLeastPrmtrN is the lowest counts to accept a pattern; PatternD records count numbers of different patterns.

1. Cut promoter sequences flanking CACGTG(…)xxxx or xxxx (…)CACGTG, which are patterns obtained in step 2.

**usage:WxW_Align.Gene PrmtrFasta Word1 Word1TH Word2 Word2TH Selected**

This script selects a subset of promoter sequences which satisfy a specified pattern in interest. The definition of PrmtrFasta, Word1, Word1TH, Word2 and Word2TH are the same as those in NearestNeighbour; Range for pattern’s distance variation is fixed in script; Selected saves promoter sequences which satisfy patterns defined by Word1 and Word2.

**usage:FlankAnchor.pl PrmtrFasta Anchor AnchorTH FlankL PrmtrFlankAnchor**

This script cuts a certain length of promoter sequences in flank of a specified pattern(determined by parameter FlankL). PrmtrFasta is the output of script WxW_Align.Gene; Anchor is Word1 of a specified pattern; AnchorTH is used to distinguish if Anchor matched; PrmtrFlankAnchor saves result in fasta format.

1. Input each group of promoter sequences obtained by FlankAnchor.pl to MEME [4] (parameters are set as: occurrences of a single motif among the sequences：any number; minimum width of each motif: 6bp; maximum width of each motif: 10bp; Maximum number of motifs to find: 5; Search given strand only), and inspect MEME’s output visually to find patterns for *cis* candidates.

Tools and databases used:

1. ftp://ftp.ncbi.nlm.nih.gov/blast/executables/blast+/LATEST/
2. ftp://ftp.ebi.ac.uk/pub/software/clustalw2/
3. http://jaspar.genereg.net/
4. http://meme.nbcr.net/meme/

***************ParseGBgz4Prdct.pl**************

#!/usr/bin/perl -w

use strict;

my $Home="/home/guanshan/";

sub Parse{

my (%List,$Path,$Src,$Dst,$File,$Cmd,%Prdct,$Locus,$Organism,$ID);

open(IN,"<$ARGV[0]") or die "Can't open $ARGV[0]!\n";

while(<IN>){$_=~/^(\S+)/; $List{$1}++;}

close(IN);

open(OUT,">$ARGV[1]") or die "Can't open $ARGV[1]!\n";

$ARGV[0]=~/^(.+\/)/; $Path=$1;

my $Processing=0;

for $File (keys %List){

print "$Processing\tProcessing $File\n"; $Processing++;

$Src=$Path.$File; $Dst=$Home."tmp/";

$Cmd="cp $Src $Dst";

system($Cmd);

$Dst.=$File;

$Cmd="gunzip $Dst";

system($Cmd);

$Dst=~s/\.gz//;

open(IN,"<$Dst") or die "Can't open $Dst!\n";

%Prdct=();

while(<IN>){

if($_=~/^LOCUS\s+(\w+)/){

$Locus=$1;

while(<IN>){

if($_=~/^\/\//){last;}

elsif($_=~/^\s+ORGANISM\s+(.+)/){$Organism="$1";}

elsif($_=~/^\s+CDS/){

$ID="File=$File Locus=$Locus Organism=$Organism";

while(<IN>){

if($_=~/^\s+\/product=\"(.+)\"?/){

$ID.=" product=\"$1\"";

$ID=~s/%//g;

}elsif($_=~/^\s+\/protein_id=\"(.+?)\"/){

$ID=$1." $ID";

}elsif($_=~/^\s+\/translation=\"(\w+)\"$/){

$Prdct{$ID}=$1; last;

}elsif($_=~/^\s+\/translation=\"(\w+)$/){

$Prdct{$ID}=$1;

while(<IN>){

if($_=~/^\s+(\w*)\"$/){

$Prdct{$ID}.=$1; last;

}elsif($_=~/^\s+(\w+)$/){

$Prdct{$ID}.=$1;

}

}

last;

}

}

}

}

}

}

close(IN);

for $ID (keys %Prdct){

printf OUT ">$ID\n";

printf OUT "$Prdct{$ID}\n";

}

$Cmd="rm -rf $Dst";

system($Cmd);

}

close(OUT);

}

# main program

@ARGV or die "Usage: ParseGBgz4Prdct.pl GenbankFileList Output\n";

Parse();

***************Blastp.pl***************

#!/usr/bin/perl -w

use strict;

# pay attentation to blast option S[1,2 or 3], Evalue and m[0,1,2,...,9]

sub formatFASTA {

my @suffix=qw(phr pin psq);

my $found=0;

print "checking for formatted database for $ARGV[1]\n";

foreach my $suffix (@suffix) {

my $file=$ARGV[1].".".$suffix;

if(!-e $file){

print "$file not found\n";

++$found;

}

}

if($found==0){

print "all files present\n";

return(1);

}

my $cmd="formatdb -i $ARGV[1] -p T";

print "executing command: $cmd\n";

system($cmd);

return(1);

}

sub doBlast {

my $cmd="blastall -p blastp -d $ARGV[1] -i $ARGV[0] -o $ARGV[2] "."-a 8 -e $ARGV[3] -b $ARGV[4] -m 8";

print "executing command: $cmd\n";

system($cmd);

}

@ARGV or die "Usage:Blastp Query Database Output eValue bValue\n";

print "blasting $ARGV[0] against $ARGV[1]\n" . "output to $ARGV[2]\n";

formatFASTA();

doBlast();

***************ParseBlastp4Hmlg.pl***************

#! /usr/bin/perl -w

use strict;

my (%Blast,%SeqLth);

sub ReadBlast_m8{

open(IN,"<$ARGV[0]")or die "Can not open $ARGV[0]\n";

while(<IN>){

$_=~/^(\S+)\s+(\S+)\s+(\S+)/;

if($3>=40){

$SeqLth{$1}=$SeqLth{$2}=0;

$Blast{$_}++;

}

}

close(IN);

}

sub ReadSeq{

my $File=shift(@_);

my (%Seq,$ID);

open(IN,"<$File") or die "Can't open $File\n";

while(<IN>){

if($_=~/^>(\S+)/){$ID=$1; $Seq{$ID}="";}

else{chomp; $Seq{$ID}.=$_;}

}

close(IN);

for $ID (keys %Seq){if(defined $SeqLth{$ID}){$SeqLth{$ID}=length($Seq{$ID});}}

}

sub ParseBlast_m8{

my (%Aln,%Map,@Tmp,$QryMatch,$SjctMatch,$QryID,$SjctID,$Qry2Sjct,$ID,$i);

for my $Blast (keys %Blast){

print $Blast;

@Tmp=split(/\s+/,$Blast);

$Qry2Sjct=$Tmp[0]." ".$Tmp[1];

if(!defined $Aln{$Qry2Sjct}){

$Aln{$Qry2Sjct}++;

$ID=$Qry2Sjct." ".$Tmp[0]; @{$Map{$ID}}=();

$ID=$Qry2Sjct." ".$Tmp[1]; @{$Map{$ID}}=();

}

$ID=$Qry2Sjct." ".$Tmp[0];

for($i=$Tmp[6];$i<=$Tmp[7];$i++){${$Map{$ID}}[$i]="x";}

$ID=$Qry2Sjct." ".$Tmp[1];

for($i=$Tmp[8];$i<=$Tmp[9];$i++){${$Map{$ID}}[$i]="x";}

}

open(OUT,">$ARGV[4]") or die "Can not open $ARGV[4]\n";

for $Qry2Sjct (keys %Aln){

print "Process $Qry2Sjct\n";

$QryMatch=$SjctMatch=0;

$Qry2Sjct=~/(\S+)\s+(\S+)/;

$QryID=$1; $SjctID=$2;

$ID=$Qry2Sjct." ".$QryID;

for($i=1;$i<=$SeqLth{$QryID};$i++){

if(defined ${$Map{$ID}}[$i] && ${$Map{$ID}}[$i] eq "x"){$QryMatch++;}

}

$ID=$Qry2Sjct." ".$SjctID;

for($i=1;$i<=$SeqLth{$SjctID};$i++){

if(defined ${$Map{$ID}}[$i] && ${$Map{$ID}}[$i] eq "x"){$SjctMatch++;}

}

$QryMatch/=$SeqLth{$QryID};

$SjctMatch/=$SeqLth{$SjctID};

if($QryMatch>=$ARGV[3]){printf OUT "$Qry2Sjct\n";}

}

close(OUT);

}

# main program

@ARGV or die "Usage:ParseBlastp4Hmlg.pl Blastp.m8 Query.Fasta Subject.Fasta Threshold Output\n";

ReadBlast_m8();

ReadSeq($ARGV[1],\%SeqLth);

ReadSeq($ARGV[2],\%SeqLth);

ParseBlast_m8();

***************ParseGBgz4Prmtr.pl***************

#!/usr/bin/perl -w

use strict;

my (%Hmlg,%File,%Locus,%Pptd,%Prmtr,%Tissue);

sub ComplementStrand{

my $PositiveRNA=shift(@_);

my ($Len,$i,$Pna,$Nna,$NegativeRNA,$Key);

$Len=length($PositiveRNA);

for($i=0;$i<$Len;$i++){

$Pna=substr($PositiveRNA, $Len-1-$i,1);

if($Pna=~/[Aa]/){$Nna="t";}

elsif($Pna=~/[Cc]/){$Nna="g";}

elsif($Pna=~/[Gg]/){$Nna="c";}

elsif($Pna=~/[Tt]/){$Nna="a";}

else{$Nna=$Pna;}

$NegativeRNA.=$Nna;

}

return $NegativeRNA;

}

sub ReadHomologFile{

open(IN,"<$ARGV[0]") or die "Can't open $ARGV[0]!\n";

while(<IN>){$_=~/^(\S+)\s+(\S+)/; $Hmlg{$1}{$2}++; $Pptd{$2}++;}

close(IN);

}

sub Read4FileList{

open(IN,"<$ARGV[1]") or die "Can't open $ARGV[1]!\n";

while(<IN>){

if($_=~/^>(\S+)\s+File=(\S+)\s+Locus=(\S+)/&&defined $Pptd{$1}){

$File{$2}++; $Locus{$3}++;

my $Pptd=$1;

if($_=~/Organism=(.+?)\s+product/){$Pptd{$Pptd}=$1;}

elsif($_=~/Organism=(.+)$/){$Pptd{$Pptd}=$1;}

}

}

close(IN);

}

sub Parse{

my ($File,$Path,$Src,$Dst,$Cmd,%Position,%CPosition,$Pptd,$Seq,$CSeq,$SeqLen,$Pos,$Start,$Len,$Strand,$Tissue);

$Path="/home/guanshan/";

for $File (keys %File){

print "processing $File ......\n";

$Src=$Path."Genbank/".$File; $Dst=$Path."tmp/";

$Cmd="cp $Src $Dst";

system($Cmd);

$Dst.=$File;

$Cmd="gunzip $Dst";

system($Cmd);

$Dst=~s/\.gz//;

open(IN,"<$Dst") or die "Can't open $Dst!\n";

while(<IN>){

if($_=~/^LOCUS\s+(\w+)/&&defined $Locus{$1}){

%Position=%CPosition=(); $Pptd=$Strand=$Pos=$Seq="";

$Tissue="tissue_type=\"unknown\"";

while(<IN>){

if($_=~/^\s+\/(tissue_type=\".+?\")/){$Tissue=$1;}

elsif($_=~/^\/\//||$_=~/^\s+CDS/){

if($Pptd ne "" && defined $Pptd{$Pptd} && $Strand ne ""&&$Pos ne ""){

# print "$Pptd\t$Strand\t$Pos\n"; <STDIN>;

if($Strand eq "+"){$Position{$Pptd}=$Pos;}

if($Strand eq "-"){$CPosition{$Pptd}=$Pos;}

$Pptd=$Strand=$Pos="";

}

if($_=~/^\s+CDS\s+join\((\d+)/||$_=~/^\s+CDS\s+<?(\d+)/){

$Strand="+"; $Pos=$1;

}elsif($_=~/^\s+CDS\s+complement/){

$Strand="-";

if($_=~/^\s+CDS\s+complement.+?\.\.(\d+)\)/){$Pos=$1;}

else{

while(<IN>){if($_=~/\.\.(\d+)\)/){$Pos=$1; last;}}

}

}

if($_=~/^\/\//){last;}

}elsif($_=~/\/protein_id=\"(.+?)\"/){

$Pptd=$1; $Tissue{$Pptd}=$Tissue;

}elsif($_=~/^ORIGIN/){$Seq="";}

elsif($_=~/^\s+\d+\s+(.+)/){chomp; $Seq.=$1;}

}

if($Seq ne ""){

$Seq=~s/\s+//g; $SeqLen=length($Seq);

if(%Position){

for $Pptd (keys %Position){

if($Position{$Pptd}>$ARGV[2]){

$Start=$Position{$Pptd}-$ARGV[3]+1;

if($Start<=0){$Start=1; $Len=$Position{$Pptd};}

else{$Len=$ARGV[3];}

$Prmtr{$Pptd}=substr($Seq,$Start-1,$Len);

}

}

}

if(%CPosition){

for $Pptd (keys %CPosition){

if($CPosition{$Pptd}+$ARGV[2]<$SeqLen){

$Start=$CPosition{$Pptd};

if($Start+$ARGV[3]<=$SeqLen){$Len=$ARGV[3];}

else{$Len=$SeqLen-$Start;}

my $tmp=substr($Seq,$Start-1,$Len);

$Prmtr{$Pptd}=ComplementStrand($tmp);

}

}

}

}

}

}

close(IN);

$Cmd="rm -rf $Dst";

system($Cmd);

}

}

sub Print{

for my $anthocyanin (keys %Hmlg){

my $Output=$anthocyanin."_".$ARGV[2]."_".$ARGV[3].".prmtr0";

open(OUT,">$Output") or die "Can't open $Output!\n";

for my $Pptd (keys %{$Hmlg{$anthocyanin}}){

if(defined $Prmtr{$Pptd}){

printf OUT ">$Pptd\tOrganism=$Pptd{$Pptd}\t$Tissue{$Pptd}\n";

printf OUT "$Prmtr{$Pptd}\n";

}

}

close(OUT);

}

}

# main program

@ARGV or die "Usage:ParseGBgz4Prmtr.pl HomologFile gbpep.fasta Least Last\n";

ReadHomologFile();

Read4FileList();

Parse();

Print();

***************CluScoreD.pl***************

#!/usr/bin/perl -w

use strict;

@ARGV or die "Usage: CluScoreD.pl Clustalw.output ScoreD\n";

my (%ScoreD,$i,$BinN,$Sum);

open(IN,"<$ARGV[0]") or die "Can't open $ARGV[0]\n";;

while(<IN>){

if($_=~/^Aligning/){

while(<IN>){

if($_=~/^Guide tree/){last;}

elsif($_=~/^Sequences.+?Score:\s+(\d+)/){$ScoreD{$1}++;}

}

}

}

close(IN);

$BinN=100;

$Sum=0;

for($i=1;$i<=$BinN;$i++){if(defined $ScoreD{$i}){$Sum+=$ScoreD{$i};}}

for($i=1;$i<=$BinN;$i++){if(defined $ScoreD{$i}){$ScoreD{$i}/=$Sum;}}

open(OUT,">$ARGV[1]") or die "Can't open $ARGV[1]\n";

for($i=1;$i<=$BinN;$i++){

if(defined $ScoreD{$i}){printf OUT "$i\t%5.3f\n",$ScoreD{$i};}

}

close(OUT);

***************CluMerge.pl***************

#!/usr/bin/perl -w

use strict;

@ARGV or die "Usage: CluMerge.pl Clustalw.output Threshold Remained\n";

my (%i2ID,%Len,%Score,$i,$j,$ID,$Flag);

open(IN,"<$ARGV[0]") or die "Can't open $ARGV[0]\n";;

while(<IN>){

if($_=~/^Sequence format is Pearson/){

while(<IN>){

if($_=~/^Start of Pairwise alignments$/){last;}

elsif($_=~/^Sequence\s+(\d+):\s+(\S+)\s+\S+\s+(\d+)/){

$i2ID{$1}=$2; $Len{$1}=$3;

}

}

}elsif($_=~/^Aligning/){

while(<IN>){

if($_=~/^Guide tree/){last;}

elsif($_=~/^Sequences\s+\((\d+):(\d+)\)\s+Aligned\.\s+Score:\s+(\d+)/){

$Score{$1}{$2}=$3;

}

}

}

}

close(IN);

$Flag=1;

while($Flag){

print "Flag=$Flag\n";

$Flag=0;

for $i (keys %i2ID){

for $j (keys %i2ID){

if($i ne $j && defined $Score{$i}{$j} && $Score{$i}{$j}>$ARGV[1]){

$Flag=1;

if($Len{$i}>=$Len{$j}){delete $i2ID{$j};}

else{delete $i2ID{$i};}

delete $Score{$i}{j};

}

}

}

}

open(OUT,">$ARGV[2]") or die "Can't open $ARGV[2]\n";

for $i (sort keys %i2ID){printf OUT "$i2ID{$i}\n";}

close(OUT);

***************NearestCluster***************

#!/usr/bin/perl -w

use strict;

my (%Prmtr,$ID,%PatternN,$d,$id);

sub Align{

my ($Qry,$Sbjt)=@_;

my ($i,$Score,@Sbjt,@Qry);

@Sbjt=split(//,$Sbjt); @Qry=split(//,$Qry);

$Score=0;

for($i=0;$i<@Sbjt;$i++){if($Sbjt[$i] eq $Qry[$i]){$Score++;}}

return $Score;

}

sub Read{

print "Reading ......\n";

open(IN,"<$ARGV[0]") or die "Can't open $ARGV[0]!\n";

while(<IN>){

chomp;

if($_=~/^>/){$ID=$_;}

else{$Prmtr{$ID}=lc($_);}

}

close(IN);

}

sub Parse{

print "Parsing ......\n";

my (%Record,$L,$W1,$W1L,$W2,$W2L,$i,$j);

$W1L=length($ARGV[1]); $W2L=length($ARGV[3]);

for $ID (keys %Prmtr){

$L=length($Prmtr{$ID});

for($i=0;$i<$L-50;$i++){

$W1=substr($Prmtr{$ID},$i,$W1L);

if(Align($W1,$ARGV[1])>=$ARGV[2]){

for($j=$i+$W1L+6;$j<$i+15&&$j<$L-40;$j++){

$W2=substr($Prmtr{$ID},$j,$W2L);

$d=$j-$i-$W1L; $id="$ARGV[1]:$W2";

$Record{$id}{$d}{$ID}++;

}

}

}

}

for $id (sort keys %Record){

for $d (keys %{$Record{$id}}){

if(keys %{$Record{$id}{$d}}>$ARGV[6]){

$PatternN{$id}{$d}=keys %{$Record{$id}{$d}};

}

}

Merge(\%{$PatternN{$id}});

}

}

sub Merge{

my $Ptr=shift(@_);

my ($i,$j,$Gap,$Min,$Left,$Right,$Middle);

do{

$Min=50; $Left=$Right=-1;

for($i=0;$i<50;$i++){

if(defined $Ptr->{$i}){

for($j=$i+1;$j<50;$j++){

if(defined $Ptr->{$j}){

if($j-$i<$Min){

$Min=$j-$i;

$Left=$i; $Right=$j;

}

last;

}

}

}

}

if($Min<$ARGV[5]){

$Gap=int(($Right+$Left)/2);

$Middle=$Ptr->{$Left}+$Ptr->{$Right};

delete $Ptr->{$Left};

delete $Ptr->{$Right};

$Ptr->{$Gap}=$Middle;

}

} while($Min<$ARGV[5]);

}

sub Print{

print "Printing ......\n";

open(OUT,">$ARGV[7]") or die "Can't open $ARGV[7]!\n";

for $id (sort keys %PatternN){

for($d=1;$d<50;$d++){

if(defined $PatternN{$id}{$d}){

print OUT ">$id:$d\t$PatternN{$id}{$d}\n";

}

}

}

close(OUT);

}

# main program

@ARGV or die "Usage:$0 PrmtrInSimpleFasta Word1 Word1TH Word2 Word2TH ClusterTH AtLeastPrmtrN PatternD\n";

Read();

Parse();

Print();

***************WxW_Align.Gene***************

#!/usr/bin/perl -w

use strict;

my ($W1L,$W2L,%Prmtr,$L,$ID,$Flag,$i,$j,$Qry,$Target,%Selected);

sub Align{

my ($Qry,$Sbjt)=@_;

my ($i,$Score,@Sbjt,@Qry);

@Sbjt=split(//,$Sbjt); @Qry=split(//,$Qry);

$Score=0;

for($i=0;$i<@Sbjt;$i++){if($Sbjt[$i] eq $Qry[$i]){$Score++;}}

return $Score;

}

sub Read{

print "Reading ......\n";

open(IN,"<$ARGV[0]") or die "Can't open $ARGV[0]!\n";

while(<IN>){

chomp;

if($_=~/^>/){$ID=$_;}

else{$Prmtr{$ID}=lc($_);}

}

close(IN);

}

sub Print{

for $ID (keys %Prmtr){

$L=length($Prmtr{$ID});

$Flag=0;

for($i=0;$i<$L-60;$i++){

$Qry=substr($Prmtr{$ID},$i,$W1L);

if(Align($Qry,$ARGV[1])>=$ARGV[2]){

$Target=uc($Qry); $Prmtr{$ID}=~s/$Qry/$Target/g; $Flag=1;

for($j=$i+$W1L+6;$j<$i+20&&$j<$L-40;$j++){

$Qry=substr($Prmtr{$ID},$j,$W2L);

if(Align($Qry,$ARGV[3])>=$ARGV[4]){

$Target=uc($Qry); $Prmtr{$ID}=~s/$Qry/$Target/g;

$Selected{$ID}++;

}

}

}

}

}

open(OUT,">$ARGV[5]") or die "Can't open $ARGV[5]!\n";

for $ID (keys %Selected){print OUT "$ID\n$Prmtr{$ID}\n";}

close(OUT);

}

# main program

@ARGV or die "Usage:$0 PrmtrInSimpleFasta Word1 Word1TH Word2 Word2TH Selected\n";

$W1L=length($ARGV[1]); $W2L=length($ARGV[3]);

Read();

Print();

***************FlankAnchor.pl***************

#!/usr/bin/perl -w

use strict;

sub Align{

my ($Qry,$Sbjt)=@_;

my ($i,$Score,@Sbjt,@Qry);

@Sbjt=split(//,$Sbjt); @Qry=split(//,$Qry);

$Score=0;

for($i=0;$i<@Sbjt;$i++){if($Sbjt[$i] eq $Qry[$i]){$Score++;}}

return $Score;

}

@ARGV or die "Usage:$0 SimpleFastaPrmtr Anchor AnchorTH FlankL PrmtrFlankAnchor\n";

my(%Seq,%SeqL,$ID,$Anchor,$i,$Start,$Len,$NewID,$Str);

open(IN,"<$ARGV[0]") or die "Can not open $ARGV[0]\n";

while(<IN>){

chomp;

if($_=~/^>/){$ID=$_;}

else{$Seq{$ID}=lc($_);}

}

close(IN);

open(OUT,">$ARGV[4]") or die "can't open $ARGV[4]\n";

for $ID (keys %Seq){

$SeqL{$ID}=length($Seq{$ID});

for($i=0;$i<$SeqL{$ID}-60;$i++){

$Anchor=substr($Seq{$ID},$i,length($ARGV[1]));

if(Align($Anchor,$ARGV[1])>=$ARGV[2]){

if($i-$ARGV[3]>0){$Start=$i-$ARGV[3];}

else{$Start=0;}

if($i+$ARGV[3]<$SeqL{$ID}){$Len=$i+$ARGV[3]-$Start+1;}

else{$Len=$SeqL{ID}-$Start+1;}

if($Len>80){

$Str=substr($Seq{$ID},$Start,$Len);

$ID=~/^(\S+)\s+(.+)$/;

$NewID=$1."_".$Start;

printf OUT "$NewID\n$Str\n";

}

}

}

}

close(OUT);
