# Supplementary material for: A valid strategy for precise identifications of transcription factor binding sites in combinatorial regulation using bioinformatic and experimental approaches
Source: Plant Methods. 2013 Aug 24;9:34. doi: 10.1186/1746-4811-9-34 (PMC3847620; doi:10.1186/1746-4811-9-34)
Supplement: Additional file 2: Table S1 — A list of 271 promoters used in the bioinformatic analysis. [file 1746-4811-9-34-S2.docx]

Additional file 2

| Table S1. **A list of 271 promoters used in the bioinformatic analysis** | | | |
| --- | --- | --- | --- |
| ID number | Promoter information | Organism | Gene used in search |
| >BAF75881.1 | nucl:AB294381.1 <1..203> | *Dianthus caryophyllus* | *3GT_b* |
| >AAB86473.1 | Nucl:KC794941 | *Ipomoea purpurea* | *3GT_b* |
| >AAA32766.1 | nucl:ATHCFI <1..661> | *Arabidopsis thaliana* | *CHI_fl1* |
| >AAA33950.1 | nucl:EF623857.1 <48797.. 49594> | *Glycine max* | *CHSD_us1* |
| >AAA33951.1 | nucl:SOYCHSVI <1..645> | *Glycine max* | *CHSD_us1* |
| >AAA67701.1 | nucl:TFRCHS6A <1..601> | *Trifolium subterraneum* | *CHSD_us1* |
| >AAA73937.1 | nucl:TFRCHS3AAA <1..601> | *Trifolium subterraneum* | *CHSD_us1* |
| >AAA73939.1 | nucl:TFRCHS5AAA <1..601> | *Trifolium subterraneum* | *CHSD_us1* |
| >AAB01004.1 | nucl:EF623856.1 <115148..115725> | *Glycine max* | *CHSD_us1* |
| >AAB20555.1 | nucl:S69616.1 <1..1340> | *Hordeum vulgare* | *DFRB_fl2* |
| >AAB58474.1 | nucl:OSU70541 <24911..26910> | *Oryza sativa* Indica Group | *DFRB_fl2* |
| >AAB94014.1 | nucl:AF010283.1 <26350..28349> | *Sorghum bicolor* | *DFRB_fl2* |
| >AAB94015.1 | nucl:AF010283.1 <37522..39521> | *Sorghum bicolor* | *DFRB_fl2* |
| >AAC18914.1 | nucl:PXU94748 <1..651> | *Petunia x hybrida* | *Ipwd1a* |
| >AAC24368.1 | nucl:CP002684.1 <360441.. 362440> | *Arabidopsis thaliana* | *CHSD_us1* |
| >AAC49030.1 | nucl:IPU15947 <1..1154> | *Ipomoea purpurea* | *CHSD_us1* |
| >AAC49929.1 | nucl:AF022142 <1..243> | *Petunia x hybrida* | *F3H_fl1* |
| >AAD21417.1 | nucl:CP002684.1 <22792757.. 22794756> | *Arabidopsis thaliana* | *DFRB_fl2* |
| >AAD26204.1 | nucl:AF117268 <1..360> | *Malus x domestica* | *DFRB_fl2* |
| >AAD41873.1 | nucl:AF152548 <1..519> | *Sorghum bicolor* | *CHSD_us1* |
| >AAD41874.1 | nucl:AF152549 <1..581> | *Sorghum bicolor* | *CHSD_us1* |
| >AAD41877.1 | nucl:AF152552 <1..461> | *Sorghum bicolor* | *CHSD_us1* |
| >AAF19756.1 | nucl:CP002684.1 <10812918..10814917> | *Arabidopsis thaliana* | *3GT_b* |
| >AAF50953.2 | nucl:AE014298.4 <21856420.. 21858419> | *Drosophila melanogaster* | *Ipwd1a* |
| >AAF71254.1 | nucl:AF227963 <1..529> | *Arachis hypogaea* | *CHSD_us1* |
| >AAF78495.1 | nucl:CP002684.1 <4396154.. 4398153> | *Arabidopsis thaliana* | *Ipwd1a* |
| >AAG25927.1 | nucl:AF260918 <1..601> | *Petunia x hybrida* | *bh2b* |
| >AAI09704.1 | nucl:BC109703.1 <1..235> | *Bos taurus* | *Ipwd1a* |
| >AAI67045.1 | nucl:NM_001107057.3 <1..229> | *Rattus norvegicus* | *Ipwd1a* |
| >AAK19620.1 | nucl:AF336287 <1..219> | *Gossypium hirsutum* | *Ipwd1a* |
| >AAK39113.1 | nucl:AF358657 <1..2000> | *Ipomoea purpurea* | *CHSD_us1* |
| >AAK39115.1 | nucl:AF358659 <2973..4972> | *Ipomoea purpurea* | *CHSD_us1* |
| >AAK92618.1 | nucl:AC078944 <143299..145298> | *Oryza sativa Japonica Group* | *F3'H_purp* |
| >AAL35830.1 | nucl:AF434703.1 <1..824> | *Triticum monococcum* | *DFRB_fl2* |
| >AAL77133.1 | nucl:AC098566.3 <133847.. 135846> | *Oryza sativa* | *CHSD_us1* |
| >AAM00948.1 | nucl:AC131968.1 <4533..6532> | *Oryza sativa* Japonica Group | *F3'H_purp* |
| >AAM13449.1 | nucl:AF474923.1 <4938..6937> | *Hordeum vulgare* subsp. vulgare | *CHI_fl1* |
| >AAM21193.1 | nucl:AF347696.1 <3531..5530> | *Zea mays* | *DFRB_fl2* |
| >AAM74394.1 | nucl:AC119149.2 <74682.. 76681> | *Oryza sativa* Japonica Group | *F3'H_purp* |
| >AAM76742.1 | nucl:AY115485.1 <1321..3320> | *Zea mays* | *Ipwd1a* |
| >AAM95644.1 | nucl:AF530910.1 <1..201> | *Gossypium hirsutum* | *Ipwd1a* |
| >AAM95646.1 | nucl:AF530912.1 <1..700> | *Gossypium hirsutum* | *Ipwd1a* |
| >AAO63026.1 | nucl:AY221250.2 <699..2698> | *Allium cepa* | *DFRB_fl2* |
| >AAO65886.1 | nucl:AC104433.8 <55507.. 57506> | *Oryza sativa* Japonica Group | *CHI_fl1* |
| >AAO67373.1 | nucl:EF623858.1 <129142.. 129977> | *Glycine max* | *CHSD_us1* |
| >AAP20865.1 | nucl:AY232493.1 <1..218> | *Anthurium andraeanum* | *F3H_fl1* |
| >AAQ62588.1 | nucl:AY262686.1 <77643.. 79642> | *Glycine max* | *CHSD_us1* |
| >AAQ62590.1 | nucl:EF623856.1 <121254..123253> | *Glycine max* | *CHSD_us1* |
| >AAQ62595.1 | nucl:EF623854.1 <42890..44889> | *Glycine max* | *CHSD_us1* |
| >AAQ62596.1 | nucl:EF623856.1 <120986.. 122985> | *Glycine max* | *CHSD_us1* |
| >AAR01949.1 | nucl:AY339884.1 <1..1445> | *Zea mays* | *Ipwd1a* |
| >AAS89833.1 | nucl:AY575057.1 <1..1442> | *Fragaria x ananassa* | *DFRB_fl2* |
| >AAS99853.1 | nucl:AY585677.2 <327..2326> | *Allium cepa* | *ANS_c* |
| >AAT44239.1 | nucl:AC136226.2 <23527.. 25526> | *Oryza sativa* Japonica Group | *CHSD_us1* |
| >AAT47094.1 | nucl:AC136226.2 <18047.. 20046> | *Oryza sativa* Japonica Group | *CHSD_us1* |
| >AAT47098.1 | nucl:AC136226.2 <45452.. 47451> | *Oryza sativa* Japonica Group | *CHSD_us1* |
| >AAT94358.1 | nucl:AY595413.1 <1..253> | *Glycine max* | *CHI_fl1* |
| >AAU00415.1 | nucl:AY566988.1 <1..295> | *Glandularia x hybrida* | *F3'H_purp* |
| >AAU04792.1 | nucl:AY691919.1 <1..446> | *Fragaria x ananassa* | *F3H_fl1* |
| >AAU12364.1 | nucl:AY695813.1 <1..1229> | *Fragaria x ananassa* | *DFRB_fl2* |
| >AAU12367.1 | nucl:AY695816.1 <1..976> | *Fragaria x ananassa* | *3GT_b* |
| >AAV70116.1 | nucl:AY825502.1 <1..619> | *Phalaenopsis hybrid* cultivar | *CHSD_us1* |
| >AAW56961.1 | nucl:AY728476.1 <7778.. 9777> | *Zea mays* | *CHSD_us1* |
| >AAW56964.1 | nucl:AY728478.1 <721..2720> | *Zea mays* | *CHSD_us1* |
| >AAX54693.1 | nucl:AY954515.1 <1..685> | *Phalaenopsis hybrid* cultivar | *CHSD_us1* |
| >AAZ79451.1 | nucl:DQ148458.1 <1..208> | *Phalaenopsis hybrid* cultivar | *F3'H_purp* |
| >AAZ80910.1 | nucl:DQ160231.1 <1..323> | *Canna generalis* | *CHI_fl1* |
| >ab006793 | nucl:AB006793.1 <4801..9146> | *Ipomoea nil* | *DFRB_fl2* |
| >ab112545 | nucl:AB112545.1 <1..2689> | *Ipomoea batatas* | *DFRB_fl2* |
| >ab232773 | nucl:AB232773.1 <1..2642> | *Ipomoea nil* | *Ipmyb1* |
| >ABB29303.1 | nucl:DQ219416.1 <6962..8961> | *Zea mays* | *DFRB_fl2* |
| >ABB84527.1 | nucl:DQ275627.2 <1..279> | *Physcomitrella patens* | *CHSD_us1* |
| >ABD04052.1 | nucl:DQ381771.1 <1..1488> | *Malus x domestica* | *ANS_c* |
| >ABF59867.1 | nucl:DQ507390.1 <1..583> | *Lupinus luteus* | *CHSD_us1* |
| >ABF82595.1 | nucl:DQ471951.1 <1..897> | *Lilium hybrid cultivar* | *CHSD_us1* |
| >ABH07784.1 | nucl:DQ834905.1 <1..1212> | *Fragaria x ananassa* | *ANS_c* |
| >ABK92281.2 | nucl:EF090266.2 <1..721> | *Polygonum cuspidatum* | *CHSD_us1* |
| >ABM64803.1 | nucl:EF187612.1 <43..1478> | *Glycine max* | *DFRB_fl2* |
| >ABM66367.1 | nucl:EF192598.1 <1091..3090> | *Allium cepa* | *ANS_c* |
| >ABN08316.1 | nucl:AC155890.2 <63363..65362> | *Medicago truncatula* | *DFRB_fl2* |
| >ABN08333.1 | nucl:AC155890.2 <117050..119049> | *Medicago truncatula* | *DFRB_fl2* |
| >ABO95764.1 | nucl:CP000584.1 <301615.. 303614> | *Ostreococcus lucimarinus CCE9901* | *Ipwd1a* |
| >ABQ63059.1 | nucl:EF623856.1 <127194..129065> | *Glycine max* | *CHSD_us1* |
| >ABR16505.1 | nucl:EF676613.1 <1..423> | *Picea sitchensis* | *Ipwd1a* |
| >ABS84871.1 | nucl:EF517133.1 <1..502> | *Linaria* sp*.* JA-2007 | *DFRB_fl2* |
| >ABV54602.1 | nucl:EF694717.1 <1..440> | *Rubus idaeus* | *CHSD_us1* |
| >ABV54603.1 | nucl:EF694718.1 <1..230> | *Rubus idaeus* | *CHSD_us1* |
| >ABW69385.1 | nucl:EU190438.1 <1..1897> | *Glycine max* | *F3'H_purp* |
| >ACC66092.1 | nucl:EU600205.1 <1..620> | *Ginkgo biloba* | *ANS_c* |
| >ACF72868.1 | nucl:EU862821.1 <1..1194> | *Rubus idaeus* | *CHSD_us1* |
| >ACG38441.1 | nucl:EU966323.1 <1..282> | *Zea mays* | *F3H_fl1* |
| >ACI33744.1 | nucl:BT045482.1 <1..373> | *Salmo salar* | *Ipwd1a* |
| >ACN31816.1 | nucl:BT065940.1 <1..645> | *Zea mays* | *CHSD_us1* |
| >ACN40174.1 | nucl:BT070669.1 <1..313> | *Picea sitchensis* | *F3'H_purp* |
| >ACO69142.1 | nucl:CP001575.1 <455396..457395> | *Micromonas* sp. RCC299 | *Ipwd1a* |
| >AY661653 | nucl:AY661653.1 <1..347> | *Snapdragon* | *Ipmyb1* |
| >BAA01512.1 | nucl:PEACHS1 <1..1571> | *Pisum sativum* | *CHSD_us1* |
| >BAA03784.1 | nucl:DARGCHS2 <431..2430> | *Daucus carota* | *CHSD_us1* |
| >BAA22042.1 | nucl:D88260.1 <1..404> | *Pisum sativum* | *CHSD_us1* |
| >BAA22043.1 | nucl:D88261.1 <1..842> | *Pisum sativum* | *CHSD_us1* |
| >BAA22044.1 | nucl:D88262.1 <1..579> | *Pisum sativum* | *CHSD_us1* |
| >BAA22076.1 | nucl:AB006793.1 <12376..14375> | *Ipomoea nil* | *DFRB_fl2* |
| >BAA36406.1 | nucl:AB011667.1 <3723..5722> | *Ipomoea purpurea* | *DFRB_fl2* |
| >BAA36407.1 | nucl:AB011667.1 <9148..11147> | *Ipomoea purpurea* | *DFRB_fl2* |
| >BAA59332.1 | nucl:AB006793.1 <175..2174> | *Ipomoea nil* | *DFRB_fl2* |
| >BAA85261.1 | nucl:AB033294.1 <595..2594> | *Arabidopsis thaliana* | *DFRB_fl2* |
| >BAA87337.1 | nucl:AB027534.1 <1..628> | *Ipomoea purpurea* | *CHSD_us1* |
| >BAA87338.1 | nucl:AB027535.1 <1..1747> | *Ipomoea nil* | *CHSD_us1* |
| >BAA87925.1 | nucl:AB022685.1 <1..241> | *Psilotum nudum* | *CHSD_us1* |
| >BAB01729.1 | nucl:CP002686.1 <9791277..9793276> | *Arabidopsis thaliana* | *Ipwd1a* |
| >BAB10427.1 | nucl:CP002688.1 <26459178..26461177> | *Arabidopsis thaliana* | *CHI_fl1* |
| >BAB11121.1 | nucl:CP002688.1 <4486763..4488762> | *Arabidopsis thaliana* | *CHSD_us1* |
| >BAB12102.1 | nucl:AB047593.2 <643..2642> | *Humulus lupulus* | *CHSD_us1* |
| >BAB61138.1 | nucl:AP003198.3 <71227.. 73226> | *Oryza sativa* Japonica Group | *ANS_c* |
| >BAB71810.1 | nucl:AB073924.1 <1..1056> | *Ipomoea nil* | *ANS_c* |
| >BAB71811.1 | nucl:AB073925.1 <5665..7664> | *Ipomoea nil* | *ANS_c* |
| >BAC97831.1 | nucl:AB078781.1 <1..1360> | *Vinca major* | *F3'H_purp* |
| >BAD00190.1 | nucl:AB113264.1 <1..1333> | *Ipomoea nil* | *F3'H_purp* |
| >BAD00191.1 | nucl:AB113266.1 <1..1308> | *Ipomoea purpurea* | *F3'H_purp* |
| >BAD00192.1 | nucl:AB113268.1 <1..1194> | *Ipomoea tricolor* | *F3'H_purp* |
| >BAD10411.1 | nucl:AP005483.3 <60681.. 62680> | *Oryza sativa* Japonica Group | *F3'H_purp* |
| >BAD18984.1 | nucl:AB154372.1 <1..922> | *Ipomoea tricolor* | *bh2b* |
| >BAD23209.1 | nucl:AP004022.3 <37975..39974> | *Oryza sativa* Japonica Group | *F3'H_purp* |
| >BAD30758.1 | nucl:AP004573.3 <112169.. 114168> | *Oryza sativa* Japonica Group | *CHSD_us1* |
| >BAD31062.1 | nucl:AP005177.4 <135141.. 137140> | *Oryza sativa* Japonica Group | *CHSD_us1* |
| >BAD36157.1 | nucl:AP005570.3 <125088.. 127087> | *Oryza sativa* Japonica Group | *F3'H_purp* |
| >BAD37752.1 | nucl:AP004737.3 <124747.. 126746> | *Oryza sativa* Japonica Group | *ANS_c* |
| >BAD38066.1 | nucl:AP005419.3 <19809..21808> | *Oryza sativa* Japonica Group | *F3'H_purp* |
| >BAD38067.1 | nucl:AP005419.3 <24767..26766> | *Oryza sativa* Japonica Group | *F3'H_purp* |
| >BAD38068.1 | nucl:AP005419.3 <41891.. 43890> | *Oryza sativa* Japonica Group | *F3'H_purp* |
| >BAD53112.1 | nucl:AP003330.4 <65846.. 67845> | *Oryza sativa* Japonica Group | *CHSD_us1* |
| >BAD89742.1 | nucl:AB199315.1 <1..679> | *Vitis vinifera* | *DFRB_fl2* |
| >BAE75806.1 | nucl:AB086055.1 <1..342> | *Vitis vinifera* | *ANS_c* |
| >BAE75809.1 | nucl:AB213565.1 <1..529> | *Vitis vinifera* | *ANS_c* |
| >BAE78769.1 | nucl:AB099529.1 <1..664> | *Agapanthus praecox* | *DFRB_fl2* |
| >BAE94710.1 | nucl:AB234212.1 <4398..6397> | *Ipomoea nil* | *Ipmyb1* |
| >BAE98273.1 | nucl:AB247917.1 <109..2108> | *Triticum aestivum* | *ANS_c* |
| >BAE98274.1 | nucl:AB247918.1 <1..1108> | *Triticum aestivum* | *ANS_c* |
| >BAE98275.1 | nucl:AB247919.1 <1..1044> | *Triticum aestivum* | *ANS_c* |
| >BAE98276.1 | nucl:AB247920.1 <1..1896> | *Triticum aestivum* | *ANS_c* |
| >BAE98277.1 | nucl:AB247921.1 <1..1641> | *Triticum aestivum* | *ANS_c* |
| >BAF08903.1 | nucl:AP004772.3 <97485.. 99484> | *Oryza sativa* Japonica Group | *Ipwd1a* |
| >BAF09665.1 | nucl:AP004178.3 <55138.. 57137> | *Oryza sativa* Japonica Group | *Ipwd1a* |
| >BAF12698.1 | nucl:AC145381.4 <51567.. 53566> | *Oryza sativa* Japonica Group | *F3'H_purp* |
| >BAF13917.1 | nucl:AL662935.3 <49127.. 51126> | *Oryza sativa* Japonica Group | *CHSD_us1* |
| >BAF15893.1 | nucl:AL606650.5 <59608..61607> | *Oryza sativa* Japonica Group | *DFRB_fl2* |
| >BAF16071.1 | nucl:AL606999.3 <57999.. 59998> | *Oryza sativa* Japonica Group | *F3H_fl1* |
| >BAF16836.1 | nucl:AC136226.2 <33660.. 35659> | *Oryza sativa* Japonica Group | *CHSD_us1* |
| >BAF19986.1 | nucl:AP003711.3 <83541.. 85540> | *Oryza sativa* Japonica Group | *F3'H_purp* |
| >BAF21101.1 | nucl:AP004002.3 <79019.. 81018> | *Oryza sativa* Japonica Group | *CHSD_us1* |
| >BAF21259.1 | nucl:AP005172.4 <21647..23646> | *Oryza sativa* Japonica Group | *CHSD_us1* |
| >BAF21633.1 | nucl:AP003995.2 <22368.. 24367> | *Oryza sativa* Japonica Group | *CHSD_us1* |
| >BAF21742.1 | nucl:AP004573.3 <76837.. 78836> | *Oryza sativa* Japonica Group | *CHSD_us1* |
| >BAF21744.1 | nucl:AP005169.3 <33839.. 35838> | *Oryza sativa* Japonica Group | *CHSD_us1* |
| >BAF24627.1 | nucl:AP006446.3 <41891.. 43890> | *Oryza sativa* Japonica Group | *F3'H_purp* |
| >BAF26111.1 | nucl:AC116600.1 <22289..24288> | *Oryza sativa* Japonica Group | *CHSD_us1* |
| >BAF26135.1 | nucl:AC131375.1 <16233..18232> | *Oryza sativa* Japonica Group | *F3'H_purp* |
| >BAF26141.1 | nucl:AC131375.1 <97154..99153> | *Oryza sativa* Japonica Group | *CHSD_us1* |
| >BAF28369.1 | nucl:AC135568.3 <141818.. 143817> | *Oryza sativa* Japonica Group | *CHSD_us1* |
| >BAF28370.1 | nucl:AC135568.3 <147817..149816> | *Oryza sativa* Japonica Group | *CHSD_us1* |
| >BAF28371.1 | nucl:AC135568.3 <158232.. 160231> | *Oryza sativa* Japonica Group | *CHSD_us1* |
| >BAF28372.1 | nucl:AC134256.4 <29294.. 31293> | *Oryza sativa* Japonica Group | *CHSD_us1* |
| >BAF28472.1 | nucl:AC133291.2 <91346..93345> | *Oryza sativa* Japonica Group | *CHSD_us1* |
| >BAF45153.1 | nucl:AB290349.1 <1..222> | *Humulus lupulus* | *DFRB_fl2* |
| >BAF46858.1 | nucl:AB252661.1 <417..2416> | *Ipomoea purpurea* | *bh2b* |
| >BAF46859.1 | nucl:AB252663.1 <1746..3745> | *Ipomoea purpurea* | *bh2b* |
| >BAF62128.1 | nucl:AB480070.1 <2828..4827> | *Glycine max* | *CHSD_us1* |
| >BAF64709.1 | nucl:AB267077.1 <2140..4139> | *Ipomoea tricolor* | *DFRB_fl2* |
| >BAF80946.1 | nucl:AB292796.1 <1..679> | *Rosa hybrid* cultivar | *3GT_b* |
| >BAG68211.1 | nucl:AB576766.1 <183..2182> | *Ipomoea batatas* | *Ipmyb1* |
| >BAH36892.1 | nucl:AB223024.1 <1..735> | *Triticum aestivum* | *F3H_fl1* |
| >BAH36893.1 | nucl:AB223025.1 <1..732> | *Triticum aestivum* | *F3H_fl1* |
| >BAH36894.1 | nucl:AB223026.1 <1..505> | *Triticum aestivum* | *F3H_fl1* |
| >BAH36903.1 | nucl:AB276087.1 <1..415> | *Aegilops tauschii x Triticum turgidum* | *DFRB_fl2* |
| >CAA03625.1 | nucl:HQ536210.1 <3680.. 3791> | *unidentified* | *DFRB_fl2* |
| >CAA27338.1 | nucl:AB691773.1 <1..1179> | *Antirrhinum majus* | *CHSD_us1* |
| >CAA32729.1 | nucl:X14589.1 <1..1425> | *Petunia x hybrida* | *CHI_fl1* |
| >CAA32730.1 | nucl:X14590.1 <1..915> | *Petunia x hybrida* | *CHI_fl1* |
| >CAA32731.1 | nucl:X14591.1 <1..1226> | *Petunia x hybrida* | *CHSD_us1* |
| >CAA32732.1 | nucl:X14592.1 <1..857> | *Petunia x hybrida* | *CHSD_us1* |
| >CAA32733.1 | nucl:X14593.1 <1..853> | *Petunia x hybrida* | *CHSD_us1* |
| >CAA32734.1 | nucl:X14594.1 <1..627> | *Petunia x hybrida* | *CHSD_us1* |
| >CAA32735.1 | nucl:X14595.1 <1..867> | *Petunia x hybrida* | *CHSD_us1* |
| >CAA32736.1 | nucl:X14596.1 <1..419> | *Petunia x hybrida* | *CHSD_us1* |
| >CAA32737.1 | nucl:X14597.1 <1..1468> | *Petunia x hybrida* | *CHSD_us1* |
| >CAA34460.1 | nucl:X16437.1 <1..955> | *Sinapis alba* | *CHSD_us1* |
| >CAA39022.1 | nucl:X55314.1 <1..1291> | *Zea mays* | *ANS_c* |
| >CAA41250.1 | nucl:X58339.1 <8..2007> | *Hordeum vulgare* | *CHSD_us1* |
| >CAA42763.1 | nucl:X60204.1 <1..512> | *Zea mays* | *CHSD_us1* |
| >CAA46590.1 | nucl:X65636.1 <1..734> | *Glycine max* | *CHSD_us1* |
| >CAA49839.1 | nucl:X70378.1 <1..604> | *Dianthus caryophyllus* | *F3H_fl1* |
| >CAA55628.1 | nucl:X78994.1 <1..1947> | *Medicago sativa* | *F3H_fl1* |
| >CAA56160.1 | nucl:X79723.1 <1..1903> | *Petunia x hybrida* | *DFRB_fl2* |
| >CAA56316.1 | nucl:X80007.1 <1..1040> | *Pisum sativum* | *CHSD_us1* |
| >CAA56317.1 | nucl:X80007.1 <2337..4336> | *Pisum sativum* | *CHSD_us1* |
| >CAA63306.1 | nucl:X92548.1 <1..1041> | *Secale cereale* | *CHSD_us1* |
| >CAA65580.1 | nucl:X96784.1 <1..950> | *Nicotiana tabacum* | *F3'H_purp* |
| >CAA75996.1 | nucl:Y16040.1 <1..678> | *Zea mays* | *DFRB_fl2* |
| >CAA78763.1 | nucl:Z15046.1 <1..673> | *Phaseolus vulgaris* | *CHI_fl1* |
| >CAA80441.1 | nucl:Z22760.1 <1..899> | *Zea mays* | *CHI_fl1* |
| >CAB45372.1 | nucl:AJ133743.1 <1382..3381> | *Arabidopsis thaliana* | *Ipwd1a* |
| >CAB45446.1 | nucl:CP002687.1 <16606350..16608349> | *Arabidopsis thaliana* | *CHSD_us1* |
| >CAB45977.1 | nucl:CP002687.1 <7309692.. 7311691> | *Arabidopsis thaliana* | *F3'H_purp* |
| >CAB62611.1 | nucl:CP002688.1 <2558438..2560437> | *Arabidopsis thaliana* | *F3'H_purp* |
| >CAB62646.1 | nucl:CP002686.1 <19023410..19025409> | *Arabidopsis thaliana* | *F3H_fl1* |
| >CAB69198.1 | nucl:AJ133743.1 <2401..2940> | *unidentified* | *Ipwd1a* |
| >CAB79243.1 | nucl:CP002687.1 <12006059.. 12008058> | *Arabidopsis thaliana* | *ANS_c* |
| >CAB80762.1 | nucl:CP002687.1 <12654..14653> | *Arabidopsis thaliana* | *CHSD_us1* |
| >CAC01716.1 | nucl:CP002688.1 <5604723.. 5606722> | *Arabidopsis thaliana* | *3GT_b* |
| >CAC01717.1 | nucl:CP002688.1 <5607476.. 5609475> | *Arabidopsis thaliana* | *3GT_b* |
| >CAC01718.1 | nucl:CP002688.1 <5609392.. 5611391> | *Arabidopsis thaliana* | *3GT_b* |
| >CAC07424.1 | nucl:AJ295838.1 <1..844> | *Populus trichocarpa* | *DFRB_fl2* |
| >CAD23044.1 | nucl:AJ430353.1 <1..1425> | *Humulus lupulus* | *CHSD_us1* |
| >CAD41695.1 | nucl:AL606650.5 <18516..20515> | *Oryza sativa* Japonica Group | *DFRB_fl2* |
| >CAE05340.2 | nucl:AL731609.2 <137936.. 139935> | *Oryza sativa* Japonica Group | *CHSD_us1* |
| >CAG32362.1 | nucl:AJ720703.1 <1..293> | *Gallus gallus* | *Ipwd1a* |
| >CAK19317.1 | nucl:AM263199.1 <1..501> | *Humulus lupulus* | *CHSD_us1* |
| >CAM27011.1 | nucl:JN961828.1 <3933..5932> | *Mus musculus* | *Ipwd1a* |
| >CAN60215.1 | nucl:AM426171.2 <4667.. 6666> | *Vitis vinifera* | *CHSD_us1* |
| >CAN60359.1 | nucl:AM429113.2 <12308..14307> | *Vitis vinifera* | *F3'H_purp* |
| >CAN61105.1 | nucl:AM450362.2 <4434.. 6433> | *Vitis vinifera* | *CHSD_us1* |
| >CAN61161.1 | nucl:AM452617.2 <8720.. 10719> | *Vitis vinifera* | *CHSD_us1* |
| >CAN61846.1 | nucl:AM472935.2 <22046.. 24045> | *Vitis vinifera* | *3GT_b* |
| >CAN61852.1 | nucl:AM473115.2 <7094..9093> | *Vitis vinifera* | *F3'H_purp* |
| >CAN61951.1 | nucl:AM477026.2 <7070.. 8429> | *Vitis vinifera* | *F3H_fl1* |
| >CAN62275.1 | nucl:AM488740.1 <4082.. 6081> | *Vitis vinifera* | *F3'H_purp* |
| >CAN62494.1 | nucl:AM427145.2 <716..2715> | *Vitis vinifera* | *CHSD_us1* |
| >CAN62671.1 | nucl:AM431358.2 <2646..4644> | *Vitis vinifera* | *CHSD_us1* |
| >CAN62848.1 | nucl:AM436565.2 <20116.. 22115> | *Vitis vinifera* | *bh2b* |
| >CAN64419.1 | nucl:AM489214.2 <4040.. 6039> | *Vitis vinifera* | *CHSD_us1* |
| >CAN66280.1 | nucl:AM479525.2 <4034.. 6033> | *Vitis vinifera* | *ANS_c* |
| >CAN66600.1 | nucl:AM423597.2 <1368..3367> | *Vitis vinifera* | *Ipwd1a* |
| >CAN68069.1 | nucl:AM463938.2 <6886.. 8885> | *Vitis vinifera* | *CHSD_us1* |
| >CAN68070.1 | nucl:AM463938.2 <16979.. 18978> | *Vitis vinifera* | *CHSD_us1* |
| >CAN68303.1 | nucl:AM471220.2 <1025..3024> | *Vitis vinifera* | *F3'H_purp* |
| >CAN68377.1 | nucl:AM473498.2 <4864..6863> | *Vitis vinifera* | *ANS_c* |
| >CAN69608.1 | nucl:AM444265.2 <8510.. 9689> | *Vitis vinifera* | *CHSD_us1* |
| >CAN70236.1 | nucl:AM461493.2 <197..2196> | *Vitis vinifera* | *F3H_fl1* |
| >CAN72347.1 | nucl:AM459972.2 <11098.. 13097> | *Vitis vinifera* | *CHSD_us1* |
| >CAN72348.1 | nucl:AM459972.2 <18080.. 20079> | *Vitis vinifera* | *CHSD_us1* |
| >CAN72728.1 | nucl:AM472190.2 <1..871> | *Vitis vinifera* | *ANS_c* |
| >CAN73761.1 | nucl:AM434691.2 <21718.. 21986> | *Vitis vinifera* | *CHSD_us1* |
| >CAN74316.1 | nucl:AM451177.2 <6245.. 8244> | *Vitis vinifera* | *CHSD_us1* |
| >CAN74526.1 | nucl:AM458653.1 <5055.. 7054> | *Vitis vinifera* | *CHI_fl1* |
| >CAN74919.1 | nucl:AM468889.2 <10943.. 11769> | *Vitis vinifera* | *3GT_b* |
| >CAN75038.1 | nucl:AM472295.2 <3027.. 4339> | *Vitis vinifera* | *CHSD_us1* |
| >CAN75347.1 | nucl:AM484178.2 <6349.. 8348> | *Vitis vinifera* | *F3'H_purp* |
| >CAN75686.1 | nucl:AM427031.2 <12280.. 14279> | *Vitis vinifera* | *F3'H_purp* |
| >CAN76086.1 | nucl:AM436933.2 <8319.. 10173> | *Vitis vinifera* | *CHSD_us1* |
| >CAN76172.1 | nucl:AM439846.2 <8224.. 10223> | *Vitis vinifera* | *CHSD_us1* |
| >CAN76937.1 | nucl:AM461096.2 <15451.. 17450> | *Vitis vinifera* | *DFRB_fl2* |
| >CAN77746.1 | nucl:AM487139.2 <5297.. 7296> | *Vitis vinifera* | *CHSD_us1* |
| >CAN77776.1 | nucl:AM488980.2 <20385.. 22384> | *Vitis vinifera* | *F3'H_purp* |
| >CAN78680.1 | nucl:AM447846.2 <645..2644> | *Vitis vinifera* | *CHSD_us1* |
| >CAN80142.1 | nucl:AM428246.2 <17818..19817> | *Vitis vinifera* | *F3'H_purp* |
| >CAN80262.1 | nucl:AM430949.2 <3835..5834> | *Vitis vinifera* | *F3H_fl1* |
| >CAN81079.1 | nucl:AM457118.1 <4921.. 6920> | *Vitis vinifera* | *F3'H_purp* |
| >CAN81926.1 | nucl:AM485293.2 <4641.. 6640> | *Vitis vinifera* | *CHSD_us1* |
| >CAN81964.1 | nucl:AM487161.2 <7741.. 9740> | *Vitis vinifera* | *CHSD_us1* |
| >CAN82000.1 | nucl:AM488451.2 <1..860> | *Vitis vinifera* | *F3'H_purp* |
| >CAN82080.1 | nucl:AM424663.2 <15459.. 17458> | *Vitis vinifera* | *CHSD_us1* |
| >CAN82569.1 | nucl:AM434769.2 <4798.. 6797> | *Vitis vinifera* | *CHSD_us1* |
| >CAN82588.1 | nucl:AM436340.2 <10263.. 12262> | *Vitis vinifera* | *F3'H_purp* |
| >CAN82604.1 | nucl:AM436584.2 <15371.. 17370> | *Vitis vinifera* | *F3'H_purp* |
| >CHSD_US1 | nucl:AF358654.1 <1..1090> | *Ipomoea purpurea* | *CHSD_us1* |
| > AAB41102.1 | nucl:AB154372.1 <3074.. 3350> | *Ipomoea purpurea* | *F3H_fl1* |
| >AAB86474.1 | nucl: nohit | *Ipomoea purpurea* | *CHI_fl1* |
| >ABW69676.1 | nucl:KC794944 | *Ipomoea purpurea* | *CHI_fl1* |
| >BAE94404.1 | nucl:AB232782.1 <4060..5094> | *Ipomoea purpurea* | *Ipwd1a* |
| >x81812 | nucl:X81812.1 <1..1881> | *Malus* sp*.* | *F3H_fl1* |
| >y13435 | nucl:Y13435.1 <1..790> | *Forsythia x intermedia* | *ANS_c* |
