# Supplementary material for: A valid strategy for precise identifications of transcription factor binding sites in combinatorial regulation using bioinformatic and experimental approaches
Source: Plant Methods. 2013 Aug 24;9:34. doi: 10.1186/1746-4811-9-34 (PMC3847620; doi:10.1186/1746-4811-9-34)
Supplement: Additional file 3: Figure S1 — Vector constructions in transient expression assays. (A) Construction of the reference and reporter vectors. The fluorescent protein-coding regions are in green. The tested promoter is shown in blue arrow. (B) Construction of the effector vectors. The TFs’ coding frames are shown in blank arrows. [file 1746-4811-9-34-S3.pptx]

## Slide 1
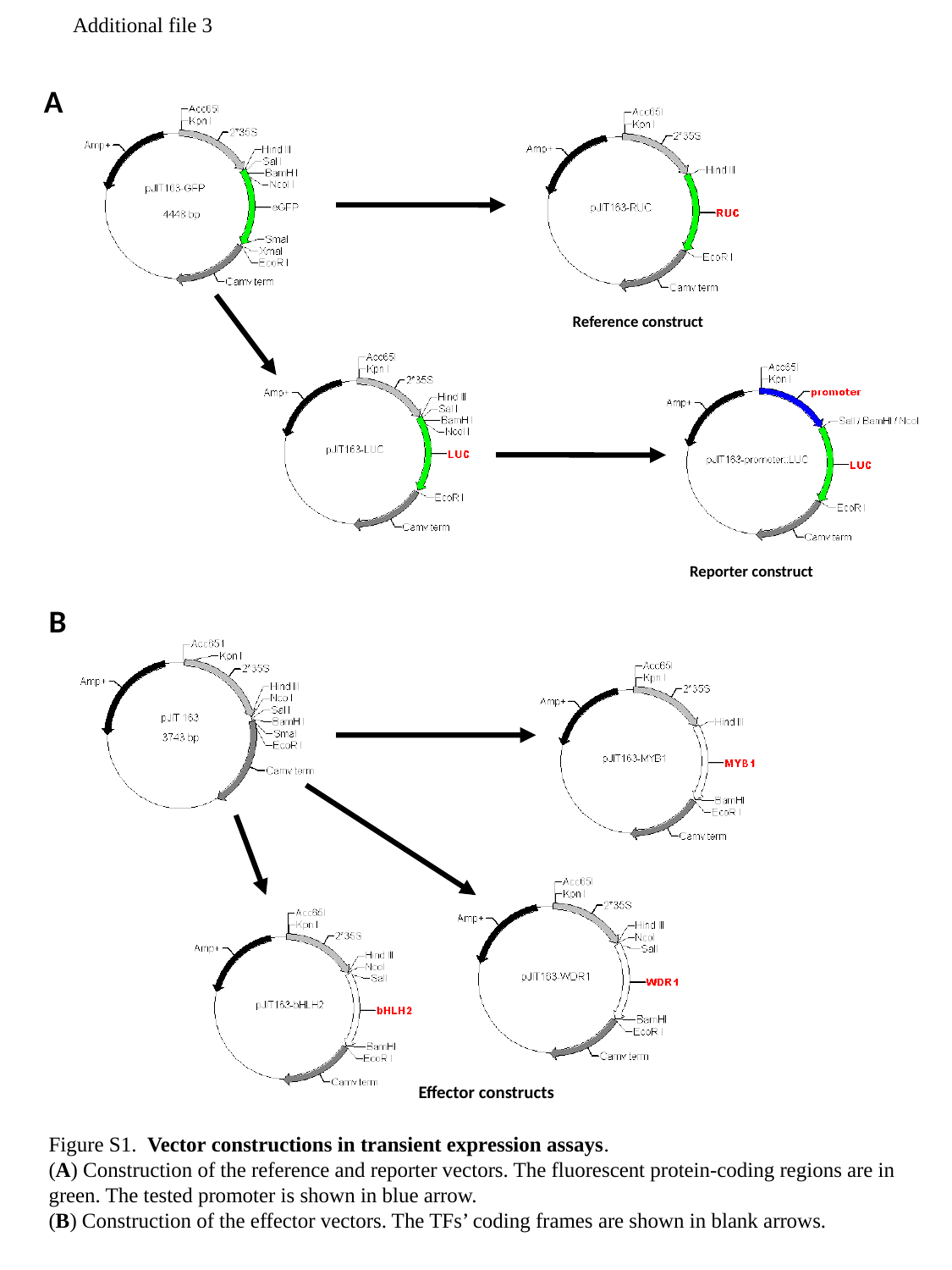

Additional file 3
A
Reference construct
Reporter construct
35S:CFP-MYB1
B
35S:YFP-bHLH2
35S:RFP-WDR1
Effector constructs
Figure S1. Vector constructions in transient expression assays.
(A) Construction of the reference and reporter vectors. The fluorescent protein-coding regions are in green. The tested promoter is shown in blue arrow.
(B) Construction of the effector vectors. The TFs’ coding frames are shown in blank arrows.
