# Supplementary material for: A valid strategy for precise identifications of transcription factor binding sites in combinatorial regulation using bioinformatic and experimental approaches
Source: Plant Methods. 2013 Aug 24;9:34. doi: 10.1186/1746-4811-9-34 (PMC3847620; doi:10.1186/1746-4811-9-34)
Supplement: Additional file 4: Figure S2 — Constructs and protein expressions for the EMSA experiments. Figure S2. Constructs and protein expressions via pMAL-c2G system for the EMSA experiments. (A) IpbHLH2 (EU032618). (B) PhAN1 (AF260919). (C) IpMYB1 (AB232769). (D) MlMYB1 (KC794950). (E) Expressed portions of the bHLHs in amino acid sequences. An EMSA test utilized the target protein expressed along with the maltose –binding protein (MBP). [file 1746-4811-9-34-S4.pptx]

## Slide 1
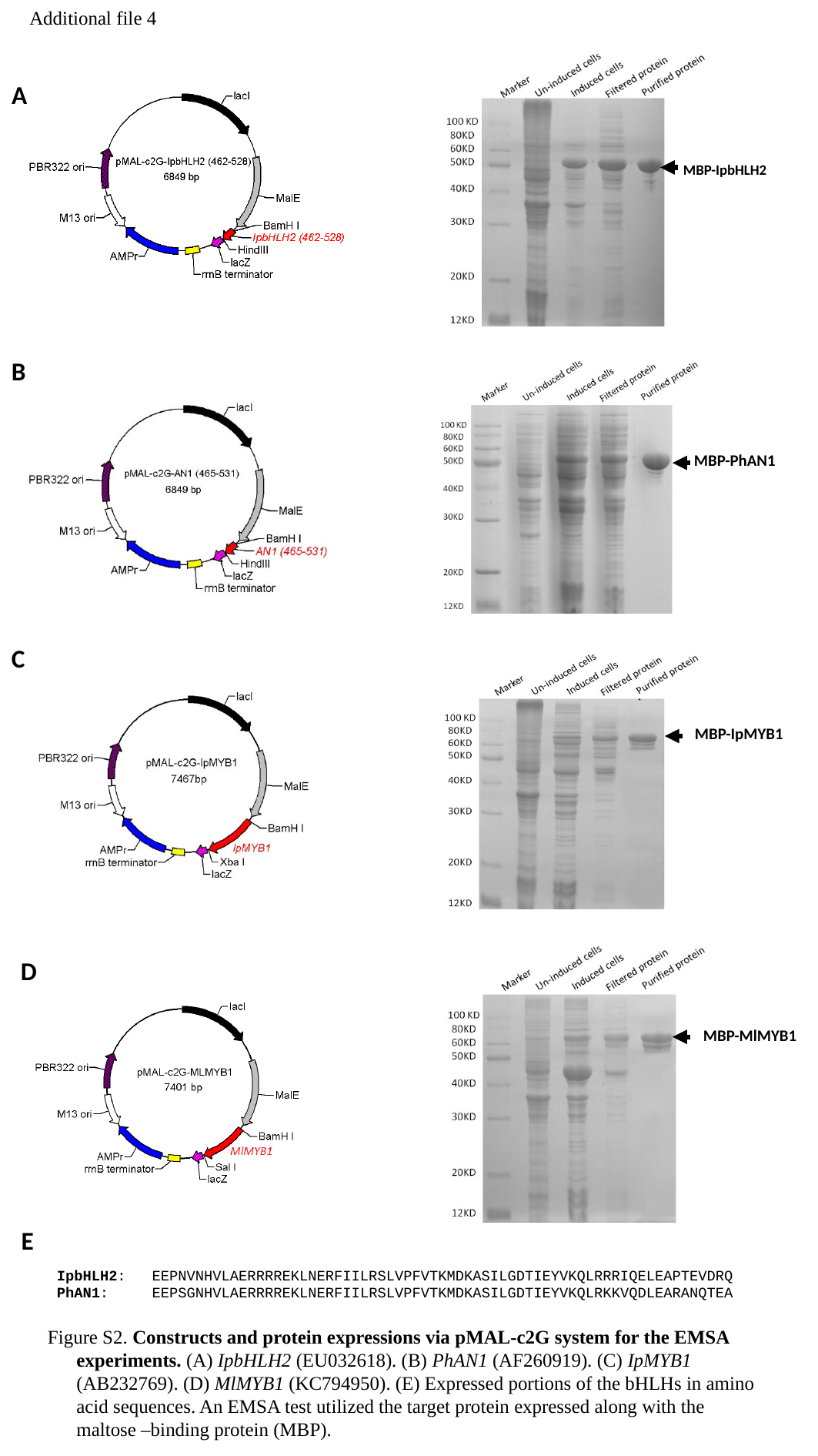

Additional file 4
MBP-IpbHLH2
A
B
MBP-PhAN1
C
MBP-IpMYB1
D
MBP-MlMYB1
E
IpbHLH2: EEPNVNHVLAERRRREKLNERFIILRSLVPFVTKMDKASILGDTIEYVKQLRRRIQELEAPTEVDRQ
PhAN1: EEPSGNHVLAERRRREKLNERFIILRSLVPFVTKMDKASILGDTIEYVKQLRKKVQDLEARANQTEA
Figure S2. Constructs and protein expressions via pMAL-c2G system for the EMSA experiments. (A) IpbHLH2 (EU032618). (B) PhAN1 (AF260919). (C) IpMYB1 (AB232769). (D) MlMYB1 (KC794950). (E) Expressed portions of the bHLHs in amino acid sequences. An EMSA test utilized the target protein expressed along with the maltose –binding protein (MBP).
